# Less continuity with more complaints: a repeated cross-sectional study of the association between relational continuity of care and patient complaints in English general practice

**DOI:** 10.1136/bmjqs-2025-018989

**Published:** 2025-10-07

**Authors:** Jinyang Chen, Panos Kasteridis, Zecharias Anteneh, Sheila Greenfield, Fiona Scheibl, Kamil Sterniczuk, Brian H Willis, Iestyn Williams, Tom Marshall

**Affiliations:** 1 Centre for Health Economics, University of York, York, UK; 2 Institute of Applied Health Research, University of Birmingham, Birmingham, UK; 3 NIHR Public Contributor, Birmingham, UK; 4 Health Services Management Centre, University of Birmingham, Birmingham, UK

**Keywords:** General practice, Patient-centred care, Patient satisfaction, Primary care, Health services research

## Abstract

**Objective:**

Relational continuity of care is associated with better patient experience and health outcomes. In England, relational continuity of primary care has been declining over a decade, coinciding with an increase in patient complaints. This study investigates the relationship between relational continuity of care and patient complaints.

**Methods:**

Cross-sectional analysis of linked practice-level data in the English National Health Service (NHS) (2016/2017–2022/2023) obtained from NHS Digital and General Practice Patient Survey (GPPS). A negative binomial model was used to investigate the association between the proportion of patients never or almost never seeing their preferred general practitioner (GP) and new written complaints per 10 000 patients, with adjustment for patient demographics, socioeconomic status, care experiences, practice care capacity and care quality. Mediation analysis was further conducted to examine patients’ lost trust and unmet clinical needs as potential mechanisms.

**Results:**

A 10 percentage point increase in the proportion of patients reporting low continuity was associated with 1.34 more new complaints per 10 000 patients (95% CI 1.23 to 1.46). The association may be stronger after than before the pandemic, among general practices with historically better continuity, and in more deprived areas. The findings were robust in using different measures of relational continuity, adjusting for primary case demand–supply mismatches, implementing a Poisson model with practice fixed effects and excluding ethnicity from the model specification. Mediation analysis showed that neither lost trust nor unmet care needs were important mediators of the effects of low continuity.

**Conclusion:**

Self-reported low continuity of primary care is associated with more patient complaints in England. Future research should explore potential underlying mechanisms and establish whether the same relationship exists between objectively measured relational continuity and patient complaints.

**WHAT IS ALREADY KNOWN ON THIS TOPIC**

- Relational continuity of care (RCC) has been steadily declining worldwide, while patient complaints have concurrently increased.

**WHAT THIS STUDY ADDS**

- In England, patient-reported decline in RCC is significantly associated with more new complaints at the practice level from 2016/2017 to 2022/2023, particularly since the pandemic, in practices with better continuity, and in more deprived areas.
- Lost trust and unmet care needs are unlikely to be the main pathways linking RCC decline and increased complaints.

**HOW THIS STUDY MIGHT AFFECT RESEARCH, PRACTICE OR POLICY**

- A continued general practitioner–patient relationship is a key determinant of patients’ experience and satisfaction.
- Micro team-based continuity of care models and their potential benefits for patients warrant further investigation.

## Introduction

 Continuity of care encompasses informational, managerial and relational continuity.^1^ Informational continuity involves sharing information between clinicians and organisations.^1^ Managerial continuity means following the same management plan across different clinicians and organisations.^1^ Both are supported and enabled by relational continuity of care (RCC), which is the ongoing clinician–patient relationship over multiple consultations.^2^

In England, both objectively measured relational continuity and subjectively reported continuity with general practitioners (GPs) are declining. An analysis of electronic health records from 100 general practices found that the Usual Provider of Care (UPC) index (ie, the proportion of a patient’s consultations with the most consulted GP decreased from 0.69 in 2006 to 0.64 in 2015.^3^ The annual General Practice Patient Survey (GPPS) asks if patients see their preferred GP.^4^ The proportion that replies ‘*Always, almost always or a lot of the time’* is a subjective measure of relational continuity, which correlates with the UPC index (Pearson correlation coefficient, 0.62), indicating a modest association between subjective and objective measures.^5^ This measure also declined from 69.8% in 2011 to 35.4% in 2023.^4^ Over the same time period, the number of written complaints in general practice increased from 42 387 in 2010/2011 to 113 041 in 2023/2024.^6^ Although complaints have been valued for reporting patients’ safety incidents and negative experiences that are less well monitored in the healthcare system,^7^ this rising trend in complaints has placed considerable stress on British doctors.^8^,^10^

A similar pattern for continuity of care and/or patient complaints has also been observed in Europe, Australia, Canada and China. In Finland, the proportion of patients seeing the same doctor dropped from around 56% in 1998 to less than half in 2011.^11^ In Ontario, Canada, a similar decline in UPC from 2004 to 2013 has also been found.^12^ In Norway, its continuity of primary care measured by the St Leonard’s Index of Continuity and Care and UPC had a slight decline prior to 2019, while the overall trend was relatively stable from 2006 to 2021.^13^ Patient complaints shared a common pattern across Australia,^14^ Canada^15^ and China,^16^^6^ where they all have experienced a consistent increase in complaints for GPs, physicians and/or the local health system over at least 5 years.

This study aims to investigate the relationship between relational continuity of primary care and patient complaint behaviour. Secondary objectives are to investigate potential mechanisms by which RCC may affect complaints and to investigate how this relationship varies across time, by the severity of discontinuity and across levels of social deprivation. The analysis is conducted at the general practice level.

## Methods

### Data source and study population

We conducted a retrospective, observational, cross-sectional study using data from the GPPS 2016/2017 to 2022/2023, several NHS Digital data sources and the Office of National Statistics (ONS). GPPS provided aggregated practice-level information on patient-reported continuity of care, demographics, long-term health conditions, appointment experiences and employment status.^4^ NHS Digital provided practice-level data on patient complaints,^6^ workforce,^17^ funding^18^ and clinical quality of care.^19^ Deprivation was measured using the Index of Multiple Deprivation (IMD) from the ONS, based on the geographical location of each practice (2015 data).^20^

The study population consisted of general practices with at least one returned patient questionnaire in the GPPS. This self-completed survey is distributed annually to over 2 million adult patients who have been registered for at least 6 months. The published patient-level response rate was 38.9% in 2016 and 29.1% in 2022.

No ethical approval was required for this study.

### Outcome, main independent variable and covariates

The outcome was the total number of new written complaints per 10 000 patients, referred to as ‘total new complaints’ for brevity. A written complaint is defined as any complaint submitted in writing to NHS staff, an NHS organisation or an NHS England region, including complaints initially made orally but later recorded in writing. NHS organisations are required to investigate written complaints and respond to the complainant. Due to data collection disruptions during the COVID-19 pandemic, complaint data for the financial year 2019/2020 were unavailable. To address this gap, the complaint data for that year were imputed using the average number of complaints in 2018/2019 and 2020/2021. We did not distinguish between complaints by subject (eg, communication, clinical issues, premises or administrative concerns), as subject data were only available at the Primary Care Trust level prior to 2016/2017.

The main independent variable was the response to the GPPS question on how often patients see or speak to their preferred GP. The responses included: ‘*I have not tried*’, ‘*Never or almost never*’, ‘*Some of the time*’, ‘*A lot of the time*’ and ‘*Always or almost always*’. In this analysis, we defined RCC as the percentage of patients in the practice who responded that they ‘*never or almost never*’ see their preferred GP. We refer to this as low continuity or discontinuity.

Potential confounders were identified based on previous literature.^521^,^24^ These included practice-level patient demographics (age, gender and ethnicity), health and healthcare-related factors (presence of long-term conditions, overall appointment experiences and waiting times), socioeconomic indicators (employment status and the English Indices of Deprivation) and primary care supply and quality metrics (GP workforce capacity, Quality and Outcomes Framework achievement, NHS payments and GP qualifications). A detailed introduction of these confounders is provided in online supplemental appendix table S1.

### Statistical analysis

The unit of analysis was the GP practice, with each practice contributing data for up to 7 years. Total new complaints is a count variable with a right-skewed, overdispersed distribution containing 3.56% zeros. Our baseline model was a negative binomial regression model with the number of registered patients (in 10 000) in each practice included as the exposure term. A Poisson regression model with practice fixed effects was estimated as a sensitivity analysis to account for unobserved, time-invariant practice-level characteristics. The Poisson fixed effects model is more robust than the negative binomial fixed effects model, which suffers from the potential incidental parameters problem.^25 26^

### Sensitivity analysis

Sensitivity analyses assessed the robustness of the findings. Two alternative measures of relational continuity were used. The first measured continuity as responses of either ‘*A lot of the time*’ or ‘*Always or almost always*’ seeing the preferred GP. The second measure was constructed by multiplying the original measure (ie, the proportion of patients who never see their preferred GP) by the proportion of patients who do have a preferred GP. In addition, mismatched demand for and supply of consultations may contribute to both worsened relational continuity and increased complaints. We assessed the effect of adjusting for this mismatch by including a proxy variable from the GPPS representing the waiting time between appointment booking and consultation (same-day, next-day, a few days later and a week or more after booking). Fourth, we excluded ethnicity as a potential confounder because missing ethnicity values resulted in additional practices being dropped from the analysis.

### Subgroup analysis

Subgroup analyses were conducted to further explore the association between relational continuity and patient complaints. The analysis was first conducted separately for each survey year, from 2016/2017 to 2022/2023. In addition, practices were categorised into quartiles based on the percentage of patients who never see their preferred GP. The first quartile represented better continuity (fewer patients reporting never seeing their preferred GP), while the fourth quartile represented worse continuity. Practices were also stratified by quintiles of the IMD 2015, with the first quintile indicating the most deprived areas and the fifth quintile the least deprived. The same modelling approach used in the main analysis was applied to these subgroup analyses.

### Mediation analysis

We employed mediation analysis^27^,^29^ to decompose the proportion of the total effect of low continuity of care on patient complaints that was potentially mediated through patients’ unmet needs (first mediator) and lack of trust and confidence (second mediator). They were measured by the responses ‘*No, not at all*’ to the GPPS questions about the patient’s most recent appointment, ‘were your needs met?’ and ‘did you have confidence and trust in the healthcare professional you saw or spoke to?’ respectively. More details can be found in online supplemental appendix table S1.

Two multivariable-adjusted models were established. The outcome model (negative binomial regression) regressed patient complaints on the percentage of patients who never see their preferred doctor, the two mediators and their interaction term. The mediator models (linear regression) regressed each mediator on the percentage of patients who never see their preferred doctor. All models were adjusted for the confounders included in the main analysis.

We decomposed the total effect of the low continuity of care on patient complaints into four components: (1) the effect mediated by unmet patient needs and lack of trust and confidence (pure indirect effect); (2) the effect from the interaction between discontinuity and the mediators (reference interaction); (3) the combined effect of mediation and interaction (mediated interaction) and (4) the direct effect, independent of mediation or interaction (controlled direct effect). These estimates were calculated by setting the mediators at their mean values and increasing low continuity from the median to the 75th percentile.

Stata V.18.0 (STATA Corporation, College Station, Texas, USA) was used for all statistical analyses.

### Patient and public involvement (PPI)

We received valuable feedback on the interpretation and presentation of our findings from a patient advisory group. One of the co-authors is a PPI co-applicant and serves as the chair of this advisory group.

### Role of the funding source

The funding source had no role in study design, data collection, data analysis, data interpretation or writing of the report.

## Results

There were 7676 eligible practices in 2016 and 6507 in 2022, yielding a total of 49 437 observations over the 7-year period from 2016 to 2022. After excluding 218 practices (about 3% of all included practices) with missing values on any included variables, the final analytical sample comprised 35 125 practice-year observations.

### Descriptive result

**Figure 1:**
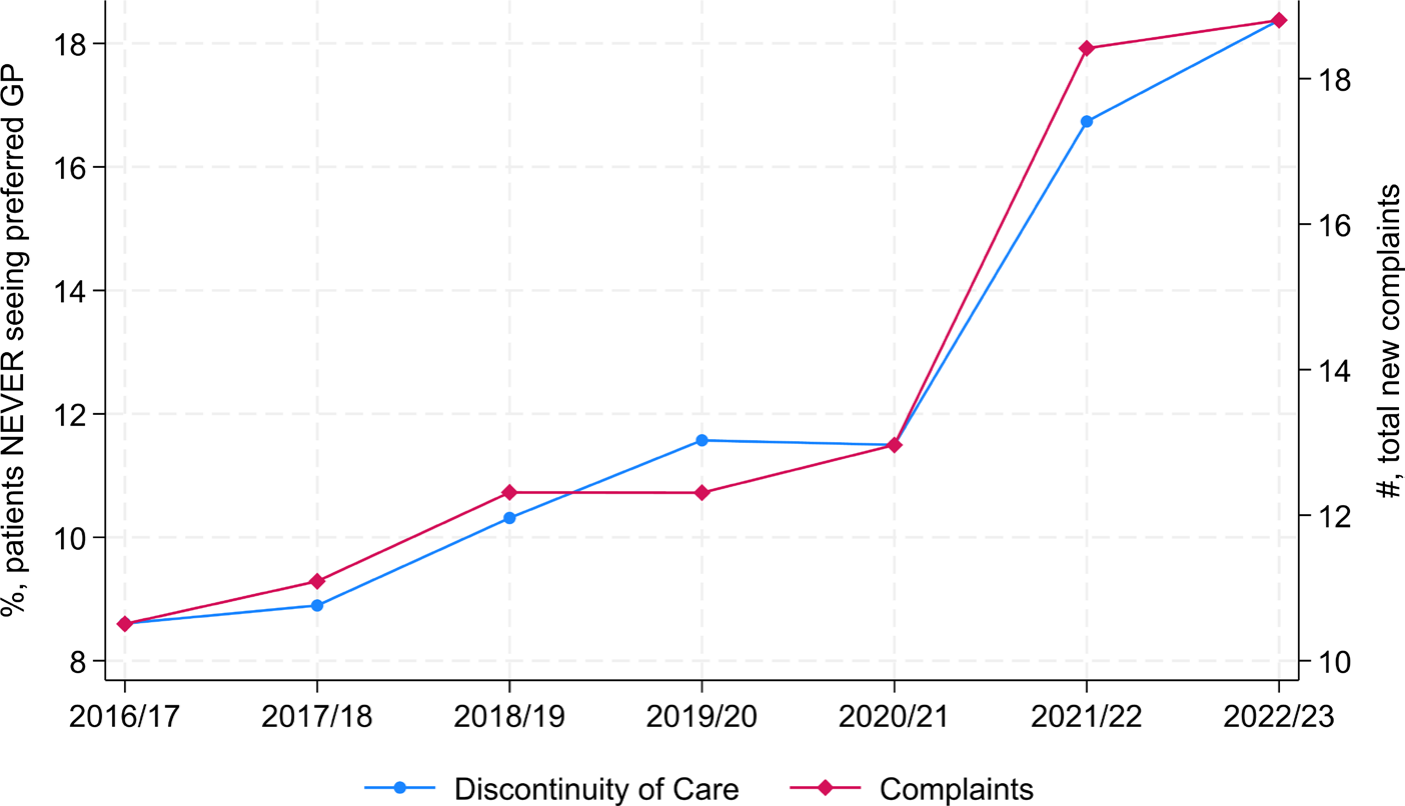
Trends in low continuity and patient complaints over time. The complaint number in 2019/2020 was imputed by the average number of complaints in 2018/2019 and 2020/2021. Continuity of care is reflected on the left Y-axis by the proportion of patients who NEVER see their preferred GP, where a higher value means lower continuity. GP, general practitioner.

Summary statistics for the outcome, the main independent variable and the covariates is provided in table 1. Between 2016/2017 and 2022/2023, an average of about 12% of patients reported that they never or almost never saw their preferred doctor. Over the same period, practices reported on average 13.76 new complaints per 10 000 patients. A consistent upward trend in both patient complaints and low continuity, with a notable increase from 2020/2021 onwards is illustrated in figure 1. Online supplemental appendix figure S1 illustrates the right-skewed distribution of both metrics in each year. The summary statistics for the variables in the mediation and sensitivity analyses are provided in online supplemental appendix table S2.

**Table 1:** Summary statistics (n=35 125)

Variables	Mean	SD	Minimum	Maximum
Total new complaints per 10 000 patients	13.76	11.75	0.00	170.48
% of patients never saw preferred doctor	12.14	10.58	0.00	77.03
% patient whose appointment experience was				
Very and fairly good	67.77	15.73	11.13	100.00
Neither good nor poor	15.85	6.33	0.00	45.82
Fairly poor	9.55	6.16	0.00	42.62
Very poor (reference category)	6.87	7.03	0.00	59.97
% of patients with long-term conditions	53.84	8.52	9.98	100.00
% male	48.30	5.71	15.50	91.26
% of patients in age group				
<65 (reference category)	76.25	8.66	37.23	100.00
65–74	12.93	4.68	0.00	33.29
75–84	7.84	3.45	0.00	24.99
>84	2.99	1.69	0.00	15.01
% of patients whose ethnicity is				
Asian	9.37	15.35	0.00	98.92
Black	3.59	6.58	0.00	64.09
Mixed	1.61	2.11	0.00	20.19
White	83.11	21.57	0.00	100.00
Other (reference category)	2.36	3.92	0.00	72.74
% of patients whose working status is				
Full or part-time work	57.32	8.23	3.36	96.08
Full-time education	4.37	5.09	0.00	94.16
Unemployed	4.42	4.04	0.00	62.02
Retired	21.84	8.67	0.00	64.20
Other (reference category)	12.12	4.94	0.00	65.41
% of patients in Index of Multiple Deprivation 2015 quintile				
1 (most deprived, reference category)	0.16			
2	0.20			
3	0.21			
4	0.22			
5 (least deprived)	0.22			
Average NHS payment per registered patient	162.48	53.61	1.80	2521.13
% of quality and outcomes framework points achieved	95.26	6.02	32.11	100.00
Number of full time GPs, per 10 000 registered patients	5.67	2.58	0.00	58.88
% of GPs with primary medical qualification from the UK	67.79	31.16	0.00	100.00

Some high values for GPs FTE per 10 000 patients reflect small patient list sizes rather than data errors. These observations were retained, as both the GP FTE and patient count values appeared valid in the original official datasets from the NHS Digital.

FTE, full-time equivalent; GPs, general practitioners; NHS, National Health Service.

### Main analysis result

**Figure 2:**
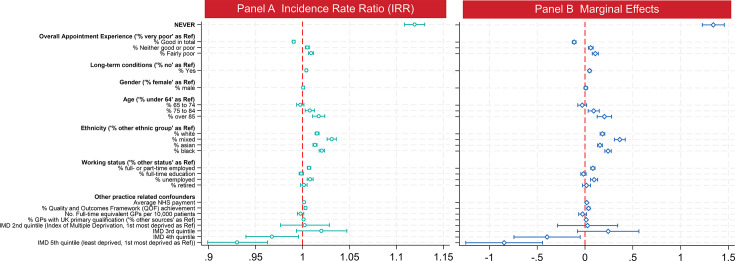
The baseline results. NEVER is the main independent variable, which represents the proportion of patients who NEVER see their preferred GP. In panel A, the X-axis represents the IRR. An IRR value of 1.1 means a 10% increase in total new written complaints per 10 000 patients at the practice level for each unit change in the independent variable. In panel B, the X-axis represents the marginal effects. A marginal effect of 1.5 means 1.5 cases increase in total new written complaints per 10 000 patients at the practice level for each unit change in the independent variable. GP, general practitioner; IRR, incidence rate ratio.

The baseline regression results as incidence rate ratios (IRR) are presented in figure 2 in panel A and marginal effects in panel B (the exact coefficients are provided in online supplemental appendix table S3). The percentage of patients never seeing their preferred doctor was rescaled by dividing by 10 to facilitate an interpretation of its coefficient as the effect of a 10 percentage point (pp) increase on total new complaints per 10 000 patients. The IRR estimate indicates that a 10 pp increase in low continuity is associated with a 1.12-fold increase (95% CI 1.11 to 1.13) in the number of new complaints. This corresponds to an increase of 1.34 new complaints per 10 000 patients (marginal effect, 95% CI 1.23 to 1.46).

### Sensitivity analysis result

Findings were similar using different measures of relational continuity, the percentage of patients who see their preferred doctor always, nearly always or a lot of the time (where higher values indicate better continuity). As shown in online supplemental appendix table S4, a 10 pp higher continuity was associated with 0.89 fewer new complaints per 10 000 patients (95% CI 0.81 to 0.96).

Using a constructed measure of discontinuity which multiplies the original measure (ie, the proportion of patients who never see their preferred GP) by the proportion of patients who do have a preferred GP, we captured the extent of unmet continuity specifically among patients with a stated preference. Compared with the original discontinuity measure, which included all patients regardless of preference, this refined variable showed a stronger association with the number of written complaints. The estimated marginal effect was 2.20 (95% CI 1.92 to 2.49), as shown in online supplemental table S5. These findings highlight that unmet expectations for relational continuity (when patients have a preferred GP but are unable to see them) are significantly associated with higher complaint rates and that the impact of discontinuity is greater when it involves patients who actively seek continuity of care.

Adjusting for waiting time did not alter the findings, as the marginal effect of low continuity remained at 1.38 (95% CI 1.27 to 1.50) in online supplemental table S6, which is very similar to the baseline estimate.

Fitting the Poisson model with practice fixed effects resulted in a lower but statistically significant marginal effect. As shown in online supplemental table S7, a 10 pp increase in the percentage of patients never seeing their preferred GP was associated with an increase of 0.55 complaints per 10 000 patients (95% CI 0.14 to 0.97). The attenuation of the effect size is expected, as the Poisson fixed-effects model accounts for unobserved, time-invariant practice-level characteristics, such as structural differences in practice management, long-term patient demographics and historical complaint patterns. This suggests that unobserved heterogeneity may have contributed to a spurious association in the main analysis, leading to a slight overestimation of the impact of low continuity on patient complaints.

Online supplemental table S8 shows that excluding ethnicity from the model specification had a small impact on the coefficient of low continuity, from 1.34 to 1.47 (95% CI 1.36 to 1.58).

### Subgroup analysis result

**Figure 3:**
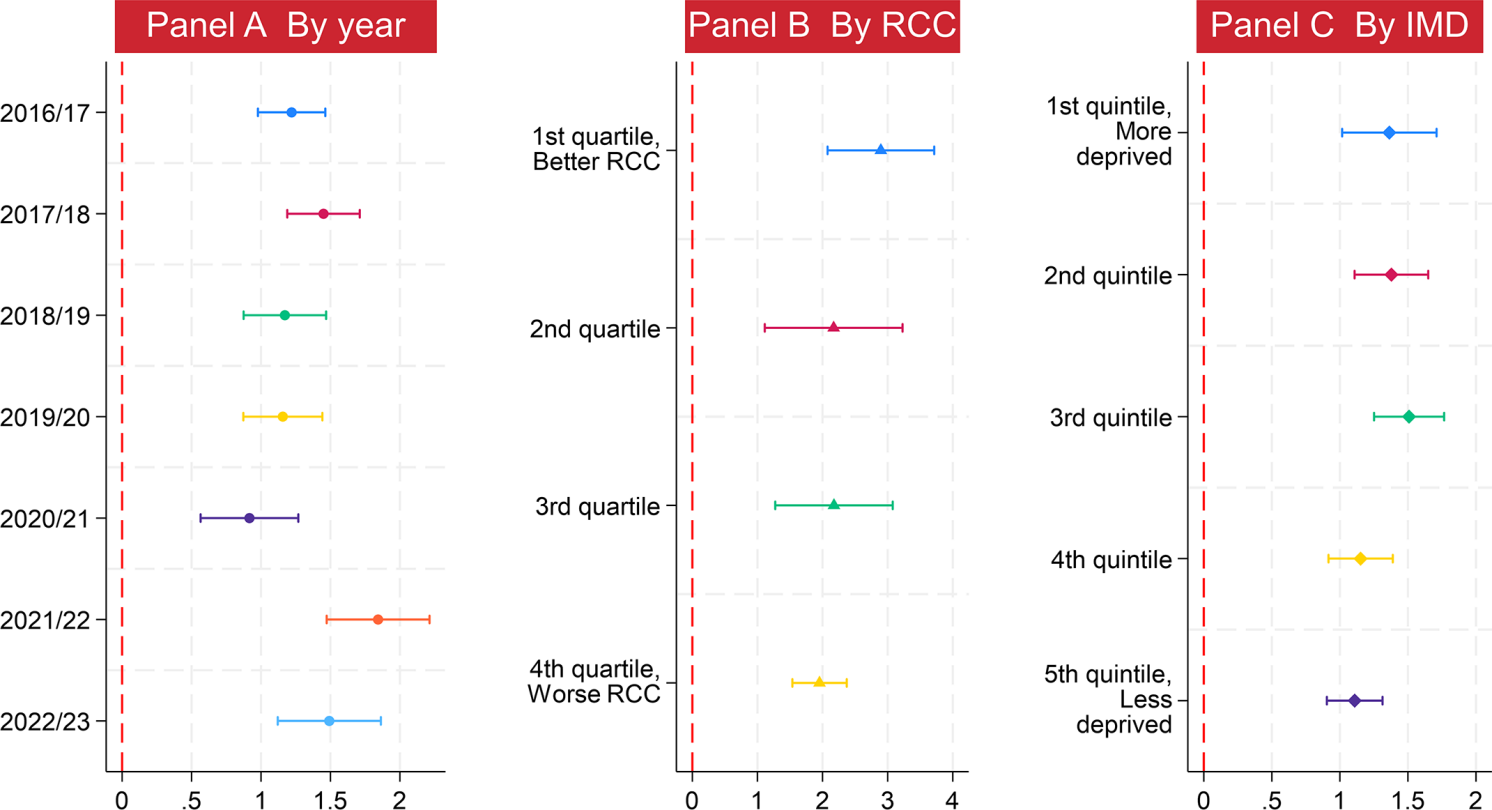
The subgroup analysis: the association between low continuity and new patient complaints. All three X-axes represent the marginal effect of the decline in relational continuity of care on patient complaints. For example, a marginal effect of 1.5 means that, on average, every 10 percentage point increase in the proportion of patients who NEVER see their preferred general practitioner is associated with a1.5-complaint increase per 10 000 patients at the practice level. IMD, the index of multiple deprivation; RCC, relational continuity of care.

The association between low continuity and new complaints per 10 000 patients was higher in postpandemic than prepandemic years. In panel A of figure 3, the marginal effects of lower continuity on complaints decreased from 1.45 (95% CI 1.19 to 1.71) in 2017/2018 to 0.92 (95% CI 0.57 to 1.27) in 2020/2021, while the strongest association (1.84, 95% CI 1.47 to 2.21) throughout the whole study period was found in 2021/2022.

The association between low continuity and patient complaints was also found to be higher in general practices with better continuity—that is, those with fewer patients never seeing their preferred GP. As shown in panel B of figure 3, a 10 pp increase in low continuity is associated with an increase in total new complaints per 10 000 patients of 2.90 (95% CI 2.08 to 3.71), 2.17 (95% CI 1.11 to 3.23), 2.18 (95% CI 1.27 to 3.08) and 1.95 (95% CI 1.54 to 2.37) for practices in the first, second, third and fourth quartiles, respectively (ie, from better to worse continuity). This suggests that patients in general practices with greater continuity of care respond more strongly to each unit decrease in continuity.

The association between low continuity and patient complaints was weaker in practices located in less deprived areas. As shown in panel C of figure 3, the estimated marginal effects of low continuity on new complaints per 10 000 patients were smaller among practices in the less deprived fourth and fifth IMD quintiles than in the more deprived first to third quintiles, particularly in terms of the clinical significance (ie, the magnitude of the coefficient).

The detailed subgroup analysis results by years and level of low continuity and deprivation scores are presented in online supplemental appendix tables S9–S11.

### Mediation analysis result

The mediation analysis shows that only 3.5% of the total effect of low continuity on complaints can be attributed to mediation by reduced patient trust and confidence (95% CI 0.025 to 0.045) and unmet needs (95% CI 0.025 to 0.046). However, the proportions explained by the controlled direct effect of patients never seeing their preferred GP (our main independent variable) were 97.9% (95% CI 0.970 to 0.987, p<0.001) when lost trust and confidence served as the mediator and 97.5% (95% CI 0.966 to 0.984, p<0.001) when unmet needs served as the mediator. These findings suggest that the rise in complaints is driven by declines in continuity of care primarily and directly (table 2). A detailed report of the mediation analysis is provided in the online supplemental appendix file S1 and online supplemental appendix table S12–S14.

**Table 2:** The four-way decomposition of the total effect of continuity of care on complaints

Mediators	Effects	Estimate	95% CI	Proportion attributed	95% CI
Lost trust and confidence	Total effect	0.091	0.082 to 0.099		
	Controlled direct effect	0.089	0.080 to 0.097	0.979	0.970 to 0.987
	Reference interaction	0.000	−0.000 to 0.000	0.001	−0.002 to 0.003
	Mediated interaction	−0.001	−0.002 to −0.001	−0.014	−0.019 to −0.009
	Pure indirect effect	0.003	0.002 to 0.004	0.035	0.025 to 0.045
Unmet clinical needs	Total effect	0.088	0.079 to 0.097		
	Controlled direct effect	0.086	0.077 to 0.095	0.975	0.966 to 0.984
	Reference interaction	0.000	−0.000 to 0.000	0.001	−0.001 to 0.003
	Mediated interaction	−0.001	−0.001 to −0.001	−0.011	−0.016 to −0.006
	Pure indirect effect	0.003	0.002 to 0.004	0.035	0.025 to 0.046

## Discussion

This study provides the first empirical evidence on the relationship between patient-reported RCC in general practice and written complaints. We found a significant, positive association between subjectively reported low continuity (never seeing preferred GP) in a general practice and rates of new written complaints per 10 000 patients from 2016/2017 to 2022/2023 in England. This relationship may have become stronger after the pandemic and may be stronger in practices in more deprived areas and where there is already high continuity. Other patient experience-related factors reported in the GPPS (lack of trust and confidence in the GP and clinical needs not being met) did not appear to mediate this relationship. These findings are robust when using different measures of continuity, controlling for the mismatch between primary care supply and demand, incorporating practice fixed effects and including practices with missing ethnicity data.

The number of patient complaints in England is not unusually high, and it is comparable with other health systems. In this study, we observed a mean of 13.76 new complaints per 10 000 patients, equivalent to about 2.75 complaints annually in a typical English general practice with 2000 patients. Similar complaint rates have been reported in other settings. For instance, a large academic medical centre in the USA recorded 12.7 complaints per 10 000 patient encounters (2017–2018),^30^ while a teaching hospital in Tokyo reported 15.6 complaints per 10 000 patients (2017–2021).^31^ In Shanghai, China, the rate of complaints across the whole local health system rose from 13.01 to 32.52 per 10 000 residents between 2017 and 2022.^16^

Our study findings are in line with previous literature. A descriptive study using qualitative thematic analysis identified a patient who cited poor continuity in contact with physicians as a reason for filing a formal complaint.^32^ Similarly, several continuity-related issues, such as unsuitable or insufficient staff, inappropriate staff conduct and distrust, were highlighted as subcategories of complaint themes in a review study aiming to develop a coding taxonomy for patient complaint analysis.^33^ Our study provides the first robust quantitative evidence supporting these qualitative findings.

Our subgroup analysis findings are also consistent with previous research on the role of expectation in shaping patient satisfaction and complaint behaviour.^34^ Patient expectation is a known predictor of satisfaction,^35 36^ and its fulfilment may influence how patients perceive and respond to changes in care quality, safety and experience. In our study, we observed that the association between low continuity and complaints was stronger in practices with greater continuity of care, particularly in the postpandemic period. We hypothesise that patients in these specific practices may have expected a return to high relational continuity after the pandemic. When these broader expectations were unmet, the decline in continuity may have been perceived more acutely, contributing to higher complaint rates, as indicated by our subgroup analyses.

The greater use of remote (telephone and video) consultation since the pandemic may not be a serious challenge to our findings. Although remote consultation was widely applied when the first national lockdown was introduced at the beginning of 2020, its usage had a clear and sharp decline trend after that.^37^ More importantly, although remote consultation was found to be risky for patient safety,^38 39^ its relationship with continuity of care was highly mixed. One review study indicated that remote consultation might be harmful for continuity of care,^40^ while another empirical analysis proved that in England, within-patient difference in continuity of primary care was actually related to the effect of the pandemic rather than directly due to the use of remote consultation.^41^ Therefore, there is no sufficient evidence suggesting that remote consultation is an important confounder in our analysis.

Our reliance on the individual-based measure of continuity (eg, how often do you see your preferred specific doctor) was, in fact, driven by the established study conventions under this topic and data restrictions. For several decades, the empirical and methodological literature on RCC has overwhelmingly focused on individual-based definitions. To ensure comparability and maintain a meaningful dialogue with this established body of work, we therefore adopted the same individual-based approach. Moreover, our study is a retrospective analysis of publicly available data, and the GPPS dataset—the primary source for our analysis—provides only individual-based measures of RCC. This means that our analysis and conclusions cannot extend to the micro team-based model of continuity that was proposed by other studies^42^ and the Royal College of General Practitioners.^43^ Future inquiry should explore the extent to which our results are repeated when applied to team-based models of continuity.

We acknowledge the following limitations to the analysis. First, the decline in the published patient-level response rate to the GPPS may affect the generalisability of the findings. However, according to the GPPS technical annexe series from 2016 to 2022, this trend is partly due to changes in sampling strategy: since 2018, the survey has boosted samples from general practices with historically lower response rates, increasing the likelihood of lower aggregate response rates. To demonstrate the impact of this sampling strategy, the GPPS also reports the weighted patient-level response rate that was adjusted for this sampling design, which has remained more stable over time (eg, from 38.9% in 2016 to 32.8% in 2022) than the unweighted and published response rate. In addition, the coverage of general practices has consistently exceeded 98%, supporting the great representativeness of the data at the practice level. These methodological features help mitigate concerns about generalisability.

Second, we do not distinguish between complaints which were upheld versus those that were not, nor between complaints related to clinical care and those concerning other aspects of general practice, due to data limitations.

Third, the GPPS continuity question might be misreported by patients. In the GPPS, it asks how often patients ‘see or speak to’ their preferred GP but does not specify whether remote consultations (eg, telephone or video) took place in the specific appointments with their preferred GP. As remote care became more common during the pandemic, patients may have varied in whether they considered such contact to represent continuity. This potential shift in interpretation may affect the measurement of continuity in the postpandemic period. The implications for our results are uncertain: if patients who under-report continuity (eg, by not counting remote contacts with their preferred GP) are also more likely to lodge complaints, the association may be overstated. However, if under-reporting occurs uniformly across patients or practices regardless of complaint behaviour, it would likely attenuate the observed relationship, underestimating the true association between continuity and complaints.

Fourth, in the mediation analysis, our two mediators (unmet clinical care needs and lost trust to health professionals) were asked about the last appointment. Therefore, it may not fully reflect the general perception of general practices’ care provision and delivery status.

Fifth, the analysis was conducted at the general practice level rather than the individual patient level. As a result, we cannot assess whether patients who personally experienced lower continuity were more likely to submit complaints, nor can we account for individual-level factors that may influence both continuity and the likelihood of complaining. This also raises the possibility of ecological bias, where associations observed at the practice level may not reflect relationships at the individual level.

Finally, it is possible that relational continuity serves as a proxy for other unmeasured practice-level characteristics that may be associated with complaint rates.

To further test the hypothesis that relational continuity influences patient complaints, future research—both quantitative and qualitative—could investigate whether individual patients’ experiences of continuity of care are associated with the frequency of written complaints. Additionally, the causal relationship between objectively measured practice-level continuity and patient complaints and the potential mechanisms warrants further investigation.

## Conclusion

This study indicates that reduced RCC in the primary care sector is significantly associated with a higher number of total new patient complaints in England. While reduced patient trust and unmet care needs are proposed as potential channels through which this relationship operates, our analysis shows that the total effect is mainly and directly from reduced RCC. This suggests that a continued GP–patient relationship remains a key determinant of patients’ primary care experience and satisfaction. In this context, the micro team-based continuity of care model and its potential benefits warrant further investigation.

## Supplementary material

10.1136/bmjqs-2025-018989 online supplemental file 1

10.1136/bmjqs-2025-018989 online supplemental file 2

10.1136/bmjqs-2025-018989 online supplemental file 3

10.1136/bmjqs-2025-018989 online supplemental file 4

10.1136/bmjqs-2025-018989 online supplemental file 5

10.1136/bmjqs-2025-018989 online supplemental file 6

10.1136/bmjqs-2025-018989 online supplemental file 7

10.1136/bmjqs-2025-018989 online supplemental file 8

## Data Availability

Data are available in a public, open access repository.

## References

[1] Reid RJ, Haggerty J, McKendry R (2002). Defusing the confusion: concepts and measures of continuity of healthcare. https://www.researchgate.net/publication/245856177.

[2] Haggerty JL, Reid RJ, Freeman GK (2003). Continuity of care: a multidisciplinary review. BMJ.

[3] El Turabi A (2019). Unintended consequences: empirical studies of continuity of care and financial incentive gaming in primary care. https://dash.harvard.edu/handle/1/41121282.

[4] GP patient survey. https://www.gp-patient.co.uk/.

[5] Hull SA, Williams C, Schofield P (2022). Measuring continuity of care in general practice: a comparison of two methods using routinely collected data. Br J Gen Pract.

[6] Data on written complaints in the NHS. https://digital.nhs.uk/data-and-information/publications/statistical/data-on-written-complaints-in-the-nhs.

[7] van Dael J, Reader TW, Gillespie A (2020). Learning from complaints in healthcare: a realist review of academic literature, policy evidence and front-line insights. BMJ Qual Saf.

[8] Bourne T, Vanderhaegen J, Vranken R (2016). Doctors’ experiences and their perception of the most stressful aspects of complaints processes in the UK: an analysis of qualitative survey data. BMJ Open.

[9] Bourne T, De Cock B, Wynants L (2017). Doctors’ perception of support and the processes involved in complaints investigations and how these relate to welfare and defensive practice: a cross-sectional survey of the UK physicians. BMJ Open.

[10] Bourne T, Wynants L, Peters M (2015). The impact of complaints procedures on the welfare, health and clinical practise of 7926 doctors in the UK: a cross-sectional survey. BMJ Open.

[11] Raivio R, Jääskeläinen J, Holmberg-Marttila D (2014). Decreasing trends in patient satisfaction, accessibility and continuity of care in Finnish primary health care - a 14-year follow-up questionnaire study. BMC Fam Pract.

[12] Singh J, Dahrouge S, Green ME (2019). The impact of the adoption of a patient rostering model on primary care access and continuity of care in urban family practices in Ontario, Canada. BMC Fam Pract.

[13] Delalic L, Grøsland M, Godager G (2024). Continuity of care in general practice in Norway. PLoS One.

[14] Parliament of Australia (2022). Administration of registration and notifications by the Australian health practitioner regulation agency and related entities under the health practitioner regulation national law. https://www.aph.gov.au/Parliamentary_Business/Committees/Senate/Community_Affairs/AHPRA/Report.

[15] College of Physicians & Surgeons of Alberta (CPSA) Complaints statistics. https://cpsa.ca/about-cpsa/statistics/.

[16] Wang G, Wu C, Yao Y (2025). Spatial-temporal analysis of patient complaints in Shanghai from 2015 to 2022. BMC Health Serv Res.

[17] General practice workforce. https://digital.nhs.uk/data-and-information/publications/statistical/general-and-personal-medical-services.

[18] NHS payments to general practice. https://digital.nhs.uk/data-and-information/publications/statistical/nhs-payments-to-general-practice.

[19] Quality and outcomes framework. https://digital.nhs.uk/data-and-information/publications/statistical/quality-and-outcomes-framework-achievement-prevalence-and-exceptions-data.

[20] English indices of deprivation 2015. https://www.gov.uk/government/statistics/english-indices-of-deprivation-2015.

[21] Tammes P, Morris RW, Murphy M (2021). Is continuity of primary care declining in England? Practice-level longitudinal study from 2012 to 2017. Br J Gen Pract.

[22] Aboulghate A, Abel G, Elliott MN (2012). Do English patients want continuity of care, and do they receive it?. Br J Gen Pract.

[23] Salant N, Massou E, Awan H (2024). Does workforce explain the relationship between funding and patient experience? A mediation analysis of primary care data in England. BMJ Open.

[24] Levene LS, Baker R, Walker N (2018). Predicting declines in perceived relationship continuity using practice deprivation scores: a longitudinal study in primary care. Br J Gen Pract.

[25] Hausman J, Hall BH, Griliches Z (1984). Econometric Models for Count Data with an Application to the Patents-R & D Relationship. Econometrica.

[26] Allison PD, Waterman RP (2002). 7. Fixed-Effects Negative Binomial Regression Models. Sociol Methodol.

[27] VanderWeele TJ (2014). A unification of mediation and interaction: a 4-way decomposition. Epidemiology.

[28] Discacciati A, Bellavia A, Lee JJ (2019). Med4way: a Stata command to investigate mediating and interactive mechanisms using the four-way effect decomposition. Int J Epidemiol.

[29] Lee JJ, Valeri L, Kapur K (2018). Growth parameters at birth mediate the relationship between prenatal manganese exposure and cognitive test scores among a cohort of 2- to 3-year-old Bangladeshi children. Int J Epidemiol.

[30] Bayer S, Kuzmickas P, Boissy A (2021). Categorizing and Rating Patient Complaints: An Innovative Approach to Improve Patient Experience. J Patient Exp.

[31] Uramatsu M, Andoh Y, Kojima T (2024). Five-year analysis of hospital complaints at a Japanese tertiary teaching hospital. Int J Qual Health Care.

[32] Råberus A, Holmström IK, Galvin K (2019). The nature of patient complaints: a resource for healthcare improvements. Int J Qual Health Care.

[33] Reader TW, Gillespie A, Roberts J (2014). Patient complaints in healthcare systems: a systematic review and coding taxonomy. BMJ Qual Saf.

[34] O’Dowd E, Lydon S, Madden C (2020). A systematic review of patient complaints about general practice. Fam Pract.

[35] Bjertnaes OA, Sjetne IS, Iversen HH (2012). Overall patient satisfaction with hospitals: effects of patient-reported experiences and fulfilment of expectations. BMJ Qual Saf.

[36] Bell RA, Kravitz RL, Thom D (2002). Unmet expectations for care and the patient-physician relationship. J Gen Intern Med.

[37] Armitage R (2023). Are video/online appointments becoming more popular among patients?. https://bjgplife.com/are-video-online-appointments-becoming-more-popular-among-patients/.

[38] Johnsen TM, Norberg BL, Kristiansen E (2021). Suitability of Video Consultations During the COVID-19 Pandemic Lockdown: Cross-sectional Survey Among Norwegian General Practitioners. J Med Internet Res.

[39] Turner A, Morris R, Rakhra D (2022). Unintended consequences of online consultations: a qualitative study in UK primary care. Br J Gen Pract.

[40] Ladds E, Khan M, Moore L (2023). The impact of remote care approaches on continuity in primary care: a mixed-studies systematic review. Br J Gen Pract.

[41] Parry W, Fraser C, Crellin E (2023). Continuity of care and consultation mode in general practice: a cross-sectional and longitudinal study using patient-level and practice-level data from before and during the COVID-19 pandemic in England. BMJ Open.

[42] Ladds E, Greenhalgh T (2023). Modernising continuity: a new conceptual framework. Br J Gen Pract.

[43] Royal College of General Practitioners (2019). RCGP guidelines for continuity of care. https://www.rcgp.org.uk/getmedia/d77f39b7-3745-4942-acef-20f4a3118c31/RCGP-continuity-of-care-guide-141119.pdf.

