# Supplementary material for: Less continuity with more complaints: a repeated cross-sectional study of the association between relational continuity of care and patient complaints in English general practice
Source: BMJ Qual Saf. 2025 Oct 7;35(6):e018989. doi: 10.1136/bmjqs-2025-018989 (PMC13217021; doi:10.1136/bmjqs-2025-018989)
Supplement: online supplemental file 1 [file bmjqs-35-6-s001.docx]

**Supplementary Appendix File S1 The result of mediation analysis**

**Outcome and mediator regression models**

The results of the multivariable-adjusted negative binomial models assessing the associations between continuity of care, two mediators (lost trust and confidence, and unmet needs), and patient complaints are presented in Supplementary Appendix Table S12. Significant interaction terms between continuity of care and both mediators—lost trust and confidence (estimate of marginal effect -0.073, 95% CI -0.093 to -0.053, *P* < 0.001) and unmet needs (estimate of marginal effect -0.061, 95% CI -0.082 to -0.041, *P* < 0.001)—suggest that the association between continuity of care and complaints is weaker in practices with higher proportions of patients reporting these concerns.

Supplementary Appendix Table S13 presents the associations between continuity of care and the two mediators separately (mediator model). Both patient’s lost trust and confidence (estimate of linear effect 0.377, 95% CI 0.329 to 0.425, *P* < 0.001) and unmet needs (estimate of linear effect 0.373, 95% CI 0.319 to 0.428, *P* < 0.001) were positively associated with a decline in continuity of care.

Supplementary Appendix Table S14 reports findings from multivariable-adjusted negative binomial models assessing continuity of care and patient complaints, with and without mediator adjustments. Adjusting for lost trust and confidence attenuated the association between continuity of care and complaints from 1.343 (95% CI 1.227 to 1.458, *P* < 0.001) to 1.172 (95% CI 1.057 to 1.288, *P* < 0.001). A similar attenuation occurred when adjusting for unmet needs (estimate of marginal effect 1.194, 95% CI 1.064 to 1.324, *P* < 0.001). When both mediators were included, the effect of reduced RCC remained stable (estimate of marginal effect 1.197, 95% CI 1.067 to 1.328, *P* < 0.001). These results suggest that lost trust and confidence, as well as unmet needs, may partially mediate the relationship between continuity of care and patient complaints, though their contribution to the total effect is limited given their relatively weak associations with continuity of care.

**A four-way decomposition of the total effect**

Table 2 presents the four-way decomposition of the total effect of continuity of care on patient complaints, with lost trust and confidence and unmet needs as mediators, each fixed at their mean values. The total effect was significant, with estimates of 0.091 (95% CI 0.082 to 0.099, *P* < 0.001) for lost trust and confidence as the mediator and 0.088 (95% CI 0.079 to 0.095, *P* < 0.001) for unmet needs as the mediator. These findings indicate that increasing the proportion of patients who never see their preferred GP from the median (9.425%) to the 75th percentile (17.314%) corresponded to a 9.1% and 8.8% increase in new written complaints per 10,000 patients, respectively.

The overall proportion of the patient’s complaints explained by the pure indirect effect (i.e., mediating effect) of patient’s lost trust (estimate of proportion attributed 0.035, 95% CI 0.025 to 0.045, *P* <0.001) and unmet needs (estimate of proportion attributed 0.035, 95% CI 0.025 to 0.046, *P* <0.001) were identical. Only 3.5% of the total effect of declined RCC on complaints could be explained by the two mediators, with the majority of the effect being direct, as shown in Table 2. The proportions attributed by the controlled direct effect of patients never seeing their preferred GP were 97.9% (95% CI 0.970 to 0.987, *P* <0.001, with lost trust and confidence as the mediator) and 97.5% (95% CI 0.966 to 0.984, *P* <0.001, with unmet needs as the mediator).

**Conclusion**

Given the minimal contributions of the pure indirect, reference interaction, and mediated interaction effects, our findings provide limited evidence that lost trust and confidence or unmet needs substantially mediate the relationship between continuity of care and patient complaints. Instead, the rise in complaints is primarily and directly driven by declines in continuity of care within primary care setting.
