# Supplementary material for: Less continuity with more complaints: a repeated cross-sectional study of the association between relational continuity of care and patient complaints in English general practice
Source: BMJ Qual Saf. 2025 Oct 7;35(6):e018989. doi: 10.1136/bmjqs-2025-018989 (PMC13217021; doi:10.1136/bmjqs-2025-018989)
Supplement: online supplemental file 2 [file bmjqs-35-6-s002.docx]

**Supplementary Appendix Table S1 Variable categories, sources, and definitions**

| **Variable Category** | **Variable Name** | **Variables Definition** | **Data Source** | **Questionnaire**  **(for GPPS variables)** | **Responses**  **(for GPPS variables)** |
| --- | --- | --- | --- | --- | --- |
| **(A) Outcome of interest** | (A.1) Complaints | Total new written complaints, **per 10,000 registered patients** | NHS Digital |  |  |
| **(B) Main independent variable** | (B.1) Continuity of care (downward measure) | % of patients who **never or almost never** see their preferred doctor | GP Patient Survey (GPPS) | How often do you see or speak to the GP you prefer? | Always or almost always  A lot of the time  Some of the time  Never or almost never  Not tried at this GP surgery |
| **(C) Confounders** | (C.1) Appointment experience | % with very and fairly good experience  % with neither good nor poor experience  % with fairly poor experience  % with very poor experience (**as reference**) | GPPS | Overall, how would you describe your experience of making an appointment? | Very good  Fairly good  Neither good nor poor  Fairly poor  Very poor |
|  | (C.2) Long-standing health conditions | % with long-term conditions  % w/o long-term conditions (**as reference**) | GPPS | Do you have a long-standing health condition? | Yes  No  Don’t know |
|  | (C.3) Employment status | % with full or part time job  % in full time education  % unemployed  % retired  % who report other status (**as reference**) | GPPS | Which of these best describes what you are doing at present? | full-time paid work (>= 30 h)  part-time paid work (< 30 h)  full-time education  unemployed  permanently sick or disabled  fully retired from work  looking after the home  doing something else |
|  | (C.4) Sex | % male  % female (**as reference**) | GPPS | Are you male or female? | Male  Female |
|  | (C.5) Ethnicity | % white ethnicity  % mixed ethnicity  % asian ethnicity  % black ethnicity  % other ethnicity (**as reference**) | GPPS | What is your ethnic group? | White  Mixed  Asian  Black  Other |
|  | (C.6) Age | % aged under 64 (**as reference**)  % aged 65-74  % aged 75-84  % aged over 85 | GPPS | How old are you? | Under 18; 18-24; 25-34; 35-44; 45-54; 55-64; 65-74; 75-84; 85 or over |
|  | (C.7) Funding | Average NHS payment per registered patient | NHS Digital |  |  |
|  | (C.8) QoF | % of quality outcome framework (QOF) points achieved | NHS Digital |  |  |
|  | (C.9) GP workforce | Total number of GP in full time equivalent, **per 10,000 registered patients** | NHS Digital |  |  |
|  | (C.10) GP qualification | % of GPs (headcount) whose primary medical qualification is from **the UK**  % of GPs whose primary medical qualification is from any other areas (**as reference**) | NHS Digital |  |  |
|  | (C.11) IMD 2015 | Quintile indicator (1^st^ represents the most deprived and 5^th^ represents the least deprived) for practice IMD (**1^st^ quintile as reference**) | Office for National Statistics |  |  |
| **(D) Variables for mediation analysis** | (D.1) Trust and confidence | % no, not at all  % yes, in total **(as reference)** | GPPS | During last general practice appointment, had confidence and trust in healthcare professional | Yes, definitely  Yes, to some extent  No, not at all  Don’t know/ Can’t say |
|  | (D.2) Patient needs | % no, not at all  % yes, in total (as reference) | GPPS | Needs met at last general practice appointment | Yes, definitely  Yes, to some extent  No, not at all  Don’t know/ Can’t say |
| **(E) Variables for sensitivity analysis** | (E.1) GP preference | % yes, in total (using base excluding ‘only one GP’) | GPPS | Have a preferred GP | Yes  Yes, for some appointments but not others  No  There is usually only one GP in my GP practice |
|  | (E.2) Continuity of care (upward measure) | % of patients who **always or almost always** see their preferred doctor (in **a lot of the time**) | GPPS | How often do you see or speak to the GP you prefer? | Always or almost always  A lot of the time  Some of the time  Never or almost never  Not tried at this GP surgery |
|  | (E.3) Waiting time | % on the same day  % on the next day  % a few days later  % a week or more later  % can’t remember (**as reference**) | GPPS | How long until the appointment took place? | On the same day  On the next day  A few days later  A week or more later  Can’t remember |
