# Supplementary material for: Less continuity with more complaints: a repeated cross-sectional study of the association between relational continuity of care and patient complaints in English general practice
Source: BMJ Qual Saf. 2025 Oct 7;35(6):e018989. doi: 10.1136/bmjqs-2025-018989 (PMC13217021; doi:10.1136/bmjqs-2025-018989)
Supplement: online supplemental file 3 [file bmjqs-35-6-s003.docx]

**Supplementary Appendix Table S2 Summary Statistics for Mediation and Sensitivity Analyses**

| **Variables** | **Number of Observations** | **Mean** | **Standard Deviation** | **Min** | **Max** |
| --- | --- | --- | --- | --- | --- |
| **Continuity of care (upward measure)** |  |  |  |  |  |
| The percentage of patients who always and almost always see their preferred GP (and in a lot of the time) | 35124 | 24.15 | 14.93 | 0.00 | 95.11 |
| **GP preference** |  |  |  |  |  |
| The percentage of patients who have a preferred GP | 35125 | 49.06 | 12.65 | 10.02 | 97.40 |
| The NET percentage of patients who NEVER see their preferred GP | 35125 | 5.34 | 4.17 | 0.00 | 35.83 |
| **Mediators** |  |  |  |  |  |
| The percentage of patients who DO NOT have trust and confidence on health professionals in their last care contact | 34908 | 5.09 | 4.12 | 0.00 | 35.44 |
| The percentage of patients whose clinical care needs are NOT met in their last care contact | 29323 | 6.49 | 4.61 | 0.00 | 32.59 |
| **Waiting time between appointment and contact (%, can’t remember as reference)** |  |  |  |  |  |
| On the same day | 35125 | 33.87 | 13.78 | 0.00 | 96.62 |
| On the next day | 35123 | 11.56 | 5.86 | 0.00 | 70.22 |
| A few days later | 35125 | 27.00 | 9.46 | 1.33 | 73.97 |
| One week or more later | 35120 | 21.48 | 11.95 | 0.00 | 77.42 |

**Note**: The NET percentage of patients who NEVER see their preferred GP is an interaction term between ‘the percentage of patients who NEVER see their preferred GP’ and ‘The percentage of patients who have a preferred GP’.
