# Supplementary material for: Less continuity with more complaints: a repeated cross-sectional study of the association between relational continuity of care and patient complaints in English general practice
Source: BMJ Qual Saf. 2025 Oct 7;35(6):e018989. doi: 10.1136/bmjqs-2025-018989 (PMC13217021; doi:10.1136/bmjqs-2025-018989)
Supplement: online supplemental file 4 [file bmjqs-35-6-s004.docx]

**Supplementary appendix table S3 Baseline results (N=35,125)**

| **Variables** | **IRR** | **95% CI** | **ME** | **95% CI** |
| --- | --- | --- | --- | --- |
| **NEVER** | 1.119^***^ | [1.109,1.130] | 1.343^***^ | [1.227,1.458] |
| **Appointment experience (%, very poor as reference)** |  |  |  |  |
| Very and fairly good | 0.991^***^ | [0.989,0.992] | -0.113^***^ | [-0.131,-0.095] |
| Neither good nor poor | 1.005^***^ | [1.003,1.007] | 0.060^***^ | [0.035,0.085] |
| Fairly poor | 1.009^***^ | [1.006,1.012] | 0.108^***^ | [0.076,0.140] |
| **Long-term Health Conditions (%, no as reference)** | 1.004^***^ | [1.003,1.005] | 0.048^***^ | [0.033,0.063] |
| Yes |  |  |  |  |
| **Gender (%, female as reference)** | 1.001 | [0.999,1.002] | 0.008 | [-0.009,0.025] |
| Male |  |  |  |  |
| **Age (%, under 64 as reference)** | 0.998 | [0.994,1.001] | -0.029 | [-0.075,0.017] |
| 65 to 74 years old | 1.008^**^ | [1.003,1.013] | 0.091^**^ | [0.033,0.149] |
| 75 to 84 years old | 1.017^***^ | [1.011,1.024] | 0.203^***^ | [0.127,0.279] |
| 85 years old and older |  |  |  |  |
| **Race (%, others as reference)** | 1.015^***^ | [1.013,1.018] | 0.183^***^ | [0.158,0.208] |
| White | 1.031^***^ | [1.026,1.036] | 0.364^***^ | [0.307,0.421] |
| Mixed | 1.013^***^ | [1.011,1.015] | 0.157^***^ | [0.131,0.182] |
| Asian | 1.020^***^ | [1.018,1.023] | 0.241^***^ | [0.208,0.275] |
| Black |  |  |  |  |
| **Working status (%, other as reference)** | 1.007^***^ | [1.005,1.009] | 0.082^***^ | [0.060,0.105] |
| Full or part-time work | 0.999 | [0.996,1.001] | -0.016 | [-0.043,0.011] |
| Full-time education | 1.008^***^ | [1.005,1.011] | 0.096^***^ | [0.058,0.133] |
| Unemployed | 1.001 | [0.998,1.005] | 0.016 | [-0.026,0.058] |
| Retired | 1.119^***^ | [1.109,1.130] | 1.343^***^ | [1.227,1.458] |
| **IMD score quintile 2015 (1^st^ quintile (most deprived) as reference)** |  |  |  |  |
| 2^nd^ quintile | 1.002 | [0.977,1.029] | 0.027 | [-0.289,0.342] |
| 3^rd^ quintile | 1.020 | [0.994,1.047] | 0.243 | [-0.079,0.564] |
| 4^th^ quintile | 0.967^*^ | [0.940,0.996] | -0.397^*^ | [-0.743,-0.050] |
| 5^th^ quintile (least deprived) | 0.930^***^ | [0.899,0.963] | -0.846^***^ | [-1.248,-0.444] |
| **Healthcare related variables** |  |  |  |  |
| Average NHS payment per registered patient | 1.002^***^ | [1.001,1.002] | 0.019^***^ | [0.017,0.022] |
| The percentage of quality outcome framework (QOF) points achieved | 1.003^***^ | [1.002,1.004] | 0.037^***^ | [0.022,0.053] |
| Total number of GP in full time equivalent, per 10,000 registered patients | 0.998 | [0.994,1.001] | -0.025 | [-0.066,0.015] |
| The percentage of GPs whose primary medical qualification is from the UK (from non-UK areas as reference) | 1.001^***^ | [1.001,1.001] | 0.013^***^ | [0.010,0.017] |

IRR, incidence rate ratio; ME, marginal effects; 95% confidence intervals in brackets; ^*^ *p* < 0.05, ^**^ *p* < 0.01, ^***^ *p* < 0.001
