# Supplementary material for: Less continuity with more complaints: a repeated cross-sectional study of the association between relational continuity of care and patient complaints in English general practice
Source: BMJ Qual Saf. 2025 Oct 7;35(6):e018989. doi: 10.1136/bmjqs-2025-018989 (PMC13217021; doi:10.1136/bmjqs-2025-018989)
Supplement: online supplemental file 5 [file bmjqs-35-6-s005.docx]

**Supplementary Appendix Table S4 Sensitivity analysis: Using alternative upward measure of continuity of care (N=35,125)**

| **Variables** | **ME** | **95% CI** |
| --- | --- | --- |
| **Always and a lot of time** | -0.885^***^ | [-0.957,-0.814] |
| **Appointment experience (%, very poor as reference)** |  |  |
| Very and fairly good | -0.120^***^ | [-0.139,-0.101] |
| Neither good nor poor | 0.017 | [-0.008,0.042] |
| Fairly poor | 0.083^***^ | [0.051,0.115] |
| **Long-term Health Conditions (%, no as reference)** |  |  |
| Yes | 0.052^***^ | [0.037,0.067] |
| **Gender (%, female as reference)** |  |  |
| Male | 0.020^*^ | [0.003,0.037] |
| **Age (%, under 64 as reference)** |  |  |
| 65 to 74 years old | -0.008 | [-0.054,0.038] |
| 75 to 84 years old | 0.103^***^ | [0.045,0.161] |
| 85 years old and older | 0.238^***^ | [0.163,0.314] |
| **Race (%, others as reference)** |  |  |
| White | 0.230^***^ | [0.206,0.255] |
| Mixed | 0.419^***^ | [0.362,0.476] |
| Asian | 0.199^***^ | [0.174,0.225] |
| Black | 0.283^***^ | [0.250,0.317] |
| **Working status (%, other as reference)** |  |  |
| Full or part-time work | 0.100^***^ | [0.078,0.122] |
| Full-time education | 0.003 | [-0.024,0.031] |
| Unemployed | 0.107^***^ | [0.069,0.144] |
| Retired | 0.016 | [-0.026,0.058] |
| **Healthcare related variables** |  |  |
| Average NHS payment per registered patient | 0.020^***^ | [0.017,0.022] |
| The percentage of quality outcome framework (QOF) points achieved | 0.039^***^ | [0.023,0.055] |
| Total number of GP in full time equivalent, per 10,000 registered patients | -0.052^*^ | [-0.093,-0.011] |
| The percentage of GPs whose primary medical qualification is from the UK (from non-UK areas as reference) | 0.013^***^ | [0.009,0.016] |
| **IMD score quintile 2015 (1^st^ quintile (most deprived) as reference)** |  |  |
| 2^nd^ quintile | 0.062 | [-0.254,0.378] |
| 3^rd^ quintile | 0.240 | [-0.081,0.562] |
| 4^th^ quintile | -0.446^*^ | [-0.792,-0.101] |
| 5^th^ quintile (least deprived) | -0.872^***^ | [-1.274,-0.469] |

Note: ME, marginal effects; 95% confidence intervals in brackets; ^*^ *p* < 0.05, ^**^ *p* < 0.01, ^***^ *p* < 0.001.

**Supplementary Appendix Table S5 Sensitivity analysis: Using the NET percentage of discontinuity (N=35,125)**

| **Variables** | **ME** | **95% CI** |
| --- | --- | --- |
| **NET NEVER** | 2.204^***^ | [1.924,2.485] |
| **Appointment experience (%, very poor as reference)** |  |  |
| Very and fairly good | -0.152^***^ | [-0.170,-0.134] |
| Neither good nor poor | 0.032^*^ | [0.007,0.057] |
| Fairly poor | 0.093^***^ | [0.061,0.125] |
| **Long-term Health Conditions (%, no as reference)** |  |  |
| Yes | 0.050^***^ | [0.035,0.065] |
| **Gender (%, female as reference)** |  |  |
| Male | 0.011 | [-0.006,0.029] |
| **Age (%, under 64 as reference)** |  |  |
| 65 to 74 years old | -0.038 | [-0.085,0.009] |
| 75 to 84 years old | 0.092^**^ | [0.033,0.151] |
| 85 years old and older | 0.204^***^ | [0.128,0.281] |
| **Race (%, others as reference)** |  |  |
| White | 0.205^***^ | [0.180,0.230] |
| Mixed | 0.392^***^ | [0.334,0.449] |
| Asian | 0.170^***^ | [0.144,0.196] |
| Black | 0.259^***^ | [0.225,0.293] |
| **Working status (%, other as reference)** |  |  |
| Full or part-time work | 0.099^***^ | [0.077,0.121] |
| Full-time education | -0.001 | [-0.029,0.026] |
| Unemployed | 0.114^***^ | [0.077,0.152] |
| Retired | 0.027 | [-0.015,0.069] |
| **Healthcare related variables** |  |  |
| Average NHS payment per registered patient | 0.020^***^ | [0.018,0.023] |
| The percentage of quality outcome framework (QOF) points achieved | 0.038^***^ | [0.022,0.054] |
| Total number of GP in full time equivalent, per 10,000 registered patients | -0.032 | [-0.073,0.010] |
| The percentage of GPs whose primary medical qualification is from the UK (from non-UK areas as reference) | 0.013^***^ | [0.010,0.016] |
| **IMD score quintile 2015 (1^st^ quintile (most deprived) as reference)** |  |  |
| 2^nd^ quintile | 0.092 | [-0.226,0.410] |
| 3^rd^ quintile | 0.330^*^ | [0.006,0.654] |
| 4^th^ quintile | -0.335 | [-0.684,0.014] |
| 5^th^ quintile (least deprived) | -0.816^***^ | [-1.222,-0.411] |

Note: ME, marginal effects; 95% confidence intervals in brackets; ^*^ *p* < 0.05, ^**^ *p* < 0.01, ^***^ *p* < 0.001.

**Supplementary Appendix Table S6 Sensitivity analysis: Adjusting for the mismatch between demand and supply (N=35,118)**

| **Variables** | **ME** | **95% CI** |
| --- | --- | --- |
| **NEVER** | 1.381^***^ | [1.265,1.498] |
| **Appointment experience (%, very poor as reference)** |  |  |
| Very and fairly good | -0.117^***^ | [-0.136,-0.098] |
| Neither good nor poor | 0.038^**^ | [0.012,0.063] |
| Fairly poor | 0.083^***^ | [0.051,0.116] |
| **Long-term Health Conditions (%, no as reference)** |  |  |
| Yes | 0.045^***^ | [0.030,0.060] |
| **Gender (%, female as reference)** |  |  |
| Male | 0.008 | [-0.009,0.025] |
| **Age (%, under 64 as reference)** |  |  |
| 65 to 74 years old | -0.031 | [-0.078,0.015] |
| 75 to 84 years old | 0.092^**^ | [0.034,0.150] |
| 85 years old and older | 0.202^***^ | [0.126,0.278] |
| **Race (%, others as reference)** |  |  |
| White | 0.171^***^ | [0.144,0.198] |
| Mixed | 0.346^***^ | [0.288,0.404] |
| Asian | 0.146^***^ | [0.118,0.174] |
| Black | 0.224^***^ | [0.188,0.260] |
| **Working status (%, other as reference)** |  |  |
| Full or part-time work | 0.073^***^ | [0.050,0.096] |
| Full-time education | -0.026 | [-0.053,0.002] |
| Unemployed | 0.093^***^ | [0.055,0.130] |
| Retired | 0.011 | [-0.030,0.053] |
| **Healthcare related variables** |  |  |
| Average NHS payment per registered patient | 0.019^***^ | [0.017,0.021] |
| The percentage of quality outcome framework (QOF) points achieved | 0.031^***^ | [0.015,0.047] |
| Total number of GP in full time equivalent, per 10,000 registered patients | -0.023 | [-0.064,0.018] |
| The percentage of GPs whose primary medical qualification is from the UK (from non-UK areas as reference) | 0.012^***^ | [0.008,0.015] |
| **IMD score quintile 2015 (1^st^ quintile (most deprived) as reference)** |  |  |
| 2^nd^ quintile | 0.053 | [-0.262,0.368] |
| 3^rd^ quintile | 0.292 | [-0.029,0.614] |
| 4^th^ quintile | -0.334 | [-0.681,0.013] |
| 5^th^ quintile (least deprived) | -0.777^***^ | [-1.180,-0.375] |
| **Waiting time (%, can’t remember as reference)** |  |  |
| Same day | 0.030^*^ | [0.005,0.055] |
| Next day | 0.030 | [-0.000,0.060] |
| A few days later | 0.030^*^ | [0.004,0.055] |
| One week after | 0.064^***^ | [0.038,0.090] |

Note: ME, marginal effects; 95% confidence intervals in brackets; ^*^ *p* < 0.05, ^**^ *p* < 0.01, ^***^ *p* < 0.001.

**Supplementary Appendix Table S7 Sensitivity analysis: Poisson model with practice fixed effects (N=34,606)**

| **Variables** | **ME** | **95% CI** |
| --- | --- | --- |
| **NEVER** | 0.554^**^ | [0.138,0.970] |
| **Appointment experience (%, very poor as reference)** |  |  |
| Very and fairly good | -0.281^***^ | [-0.427,-0.134] |
| Neither good nor poor | -0.154^***^ | [-0.239,-0.070] |
| Fairly poor | -0.086^**^ | [-0.149,-0.023] |
| **Long-term Health Conditions (%, no as reference)** |  |  |
| Yes | 0.018 | [-0.014,0.051] |
| **Gender (%, female as reference)** |  |  |
| Male | -0.009 | [-0.035,0.016] |
| **Age (%, under 64 as reference)** |  |  |
| 65 to 74 years old | -0.019 | [-0.091,0.053] |
| 75 to 84 years old | 0.138^*^ | [0.006,0.270] |
| 85 years old and older | 0.016 | [-0.119,0.152] |
| **Race (%, others as reference)** |  |  |
| White | 0.003 | [-0.066,0.073] |
| Mixed | -0.013 | [-0.120,0.095] |
| Asian | 0.010 | [-0.071,0.090] |
| Black | -0.013 | [-0.099,0.073] |
| **Working status (%, other as reference)** |  |  |
| Full or part-time work | -0.036 | [-0.079,0.007] |
| Full-time education | -0.002 | [-0.056,0.052] |
| Unemployed | -0.010 | [-0.074,0.053] |
| Retired | -0.056 | [-0.135,0.023] |
| **Healthcare related variables** |  |  |
| Average NHS payment per registered patient | 0.018^*^ | [0.002,0.034] |
| The percentage of quality outcome framework (QOF) points achieved | -0.040 | [-0.082,0.002] |
| Total number of GP in full time equivalent, per 10,000 registered patients | 0.051 | [-0.087,0.188] |
| The percentage of GPs whose primary medical qualification is from the UK (from non-UK areas as reference) | -0.006 | [-0.018,0.006] |

Note: ME, marginal effects; 95% confidence intervals in brackets; ^*^ *p* < 0.05, ^**^ *p* < 0.01, ^***^ *p* < 0.001.

**Supplementary Appendix Table S8 Sensitivity analysis: Excluding ethnicity (N=40,408)**

| **Variables** | **ME** | **95% CI** |
| --- | --- | --- |
| **NEVER** | 1.470^***^ | [1.364,1.575] |
| **Appointment experience (%, very poor as reference)** |  |  |
| Very and fairly good | -0.067^***^ | [-0.083,-0.051] |
| Neither good nor poor | 0.105^***^ | [0.083,0.127] |
| Fairly poor | 0.176^***^ | [0.147,0.204] |
| **Long-term Health Conditions (%, no as reference)** |  |  |
| Yes | 0.086^***^ | [0.073,0.098] |
| **Gender (%, female as reference)** |  |  |
| Male | 0.004 | [-0.012,0.019] |
| **Age (%, under 64 as reference)** |  |  |
| 65 to 74 years old | -0.014 | [-0.057,0.030] |
| 75 to 84 years old | 0.111^***^ | [0.056,0.165] |
| 85 years old and older | 0.215^***^ | [0.144,0.286] |
| **Working status (%, other as reference)** |  |  |
| Full or part-time work | 0.187^***^ | [0.170,0.205] |
| Full-time education | 0.078^***^ | [0.055,0.101] |
| Unemployed | 0.165^***^ | [0.131,0.198] |
| Retired | 0.085^***^ | [0.047,0.122] |
| **Healthcare related variables** |  |  |
| Average NHS payment per registered patient | 0.021^***^ | [0.019,0.023] |
| The percentage of quality outcome framework (QOF) points achieved | 0.059^***^ | [0.044,0.073] |
| Total number of GP in full time equivalent, per 10,000 registered patients | -0.019 | [-0.057,0.019] |
| The percentage of GPs whose primary medical qualification is from the UK (from non-UK areas as reference) | 0.015^***^ | [0.012,0.018] |
| **IMD score quintile 2015 (1^st^ quintile (most deprived) as reference)** |  |  |
| 2^nd^ quintile | 0.132 | [-0.160,0.424] |
| 3^rd^ quintile | 0.401^**^ | [0.105,0.698] |
| 4^th^ quintile | -0.112 | [-0.427,0.204] |
| 5^th^ quintile (least deprived) | -0.084 | [-0.447,0.279] |

Note: ME, marginal effects; 95% confidence intervals in brackets; ^*^ *p* < 0.05, ^**^ *p* < 0.01, ^***^ *p* < 0.001.
