# Supplementary material for: Less continuity with more complaints: a repeated cross-sectional study of the association between relational continuity of care and patient complaints in English general practice
Source: BMJ Qual Saf. 2025 Oct 7;35(6):e018989. doi: 10.1136/bmjqs-2025-018989 (PMC13217021; doi:10.1136/bmjqs-2025-018989)
Supplement: online supplemental file 6 [file bmjqs-35-6-s006.docx]

**Supplementary Appendix Table S9.1 Subgroup analysis results: By year (2016/17 to 2018/19, to be continued)**

| **Variables** | **2016/17**  **(N=5,714)** | | **2017/18**  **(N=5,588)** | | **2018/19**  **(N=4,405)** | |
| --- | --- | --- | --- | --- | --- | --- |
|  | **ME** | **95% CI** | **ME** | **95% CI** | **ME** | **95% CI** |
| **NEVER** | 1.220^***^ | [0.978,1.463] | 1.450^***^ | [1.189,1.711] | 1.172^***^ | [0.875,1.469] |
| **Appointment experience (%, very poor as reference)** |  |  |  |  |  |  |
| Very and fairly good | -0.051^*^ | [-0.093,-0.010] | -0.090^***^ | [-0.134,-0.045] | -0.122^***^ | [-0.179,-0.066] |
| Neither good nor poor | 0.138^***^ | [0.082,0.194] | 0.023 | [-0.036,0.083] | 0.030 | [-0.043,0.103] |
| Fairly poor | 0.138^***^ | [0.068,0.208] | 0.096^*^ | [0.023,0.170] | 0.095^*^ | [0.006,0.184] |
| **Long-term Health Conditions (%, no as reference)** |  |  |  |  |  |  |
| Yes | 0.042^**^ | [0.010,0.074] | 0.061^***^ | [0.030,0.092] | 0.020 | [-0.017,0.058] |
| **Gender (%, female as reference)** |  |  |  |  |  |  |
| Male | 0.010 | [-0.027,0.046] | -0.022 | [-0.058,0.015] | -0.002 | [-0.044,0.040] |
| **Age (%, under 64 as reference)** |  |  |  |  |  |  |
| 65 to 74 years old | -0.024 | [-0.122,0.075] | 0.043 | [-0.052,0.137] | -0.036 | [-0.153,0.082] |
| 75 to 84 years old | -0.066 | [-0.186,0.054] | -0.054 | [-0.173,0.066] | 0.011 | [-0.136,0.158] |
| 85 years old and older | 0.091 | [-0.051,0.233] | 0.182^*^ | [0.032,0.332] | 0.322^***^ | [0.132,0.512] |
| **Race (%, others as reference)** |  |  |  |  |  |  |
| White | 0.125^***^ | [0.076,0.175] | 0.129^***^ | [0.071,0.186] | 0.179^***^ | [0.110,0.248] |
| Mixed | 0.221^**^ | [0.087,0.355] | 0.334^***^ | [0.209,0.460] | 0.282^***^ | [0.138,0.427] |
| Asian | 0.080^**^ | [0.025,0.136] | 0.111^***^ | [0.056,0.166] | 0.150^***^ | [0.078,0.222] |
| Black | 0.168^***^ | [0.097,0.239] | 0.198^***^ | [0.122,0.275] | 0.248^***^ | [0.155,0.342] |
| **Working status (%, other as reference)** |  |  |  |  |  |  |
| Full or part-time work | 0.038 | [-0.007,0.084] | 0.097^***^ | [0.052,0.141] | 0.068^*^ | [0.011,0.126] |
| Full-time education | -0.034 | [-0.087,0.020] | 0.008 | [-0.049,0.065] | -0.008 | [-0.073,0.057] |
| Unemployed | 0.055 | [-0.020,0.130] | 0.113^**^ | [0.032,0.195] | 0.097 | [-0.006,0.200] |
| Retired | 0.014 | [-0.069,0.097] | 0.051 | [-0.032,0.135] | 0.027 | [-0.080,0.133] |
| **Healthcare related variables** |  |  |  |  |  |  |
| Average NHS payment per registered patient | 0.015^***^ | [0.008,0.021] | 0.016^***^ | [0.012,0.021] | 0.020^***^ | [0.015,0.026] |
| The percentage of quality outcome framework (QOF) points achieved | 0.033^*^ | [0.003,0.063] | 0.017 | [-0.018,0.051] | 0.051^*^ | [0.007,0.094] |
| Total number of GP in full time equivalent, per 10,000 registered patients | -0.070 | [-0.163,0.022] | -0.068 | [-0.170,0.035] | -0.072 | [-0.170,0.027] |
| The percentage of GPs whose primary medical qualification is from the UK (from non-UK areas as reference) | 0.008^*^ | [0.002,0.015] | 0.014^***^ | [0.007,0.021] | 0.016^***^ | [0.007,0.025] |
| **IMD score quintile 2015 (1^st^ quintile (most deprived) as reference)** |  |  |  |  |  |  |
| 2^nd^ quintile | 0.511 | [-0.107,1.129] | 0.383 | [-0.217,0.982] | -0.025 | [-0.723,0.672] |
| 3^rd^ quintile | 0.260 | [-0.350,0.869] | 0.660^*^ | [0.056,1.265] | 0.726 | [-0.053,1.506] |
| 4^th^ quintile | 0.162 | [-0.487,0.812] | 0.223 | [-0.453,0.900] | -0.007 | [-0.823,0.810] |
| 5^th^ quintile (least deprived) | -0.276 | [-1.059,0.507] | 0.115 | [-0.648,0.878] | -0.426 | [-1.423,0.572] |

ME, marginal effects; 95% confidence intervals in brackets; ^*^ *p* < 0.05, ^**^ *p* < 0.01, ^***^ *p* < 0.001.

**Supplementary Appendix Table S9.2 Subgroup analysis results: By year (2020/21 to 2022/23)**

| **Variables** | **2019/20**  **(N=4,808)** | | **2020/21**  **(N=4,943)** | | **2021/22**  **(N=4,829)** | | **2022/23**  **(N=4,838)** | |
| --- | --- | --- | --- | --- | --- | --- | --- | --- |
|  | **ME** | **95% CI** | **ME** | **95% CI** | **ME** | **95% CI** | **ME** | **95% CI** |
| **NEVER** | 1.157^***^ | [0.873,1.441] | 0.917^***^ | [0.565,1.269] | 1.843^***^ | [1.473,2.213] | 1.492^***^ | [1.120,1.863] |
| **Appointment experience (%, very poor as reference)** |  |  |  |  |  |  |  |  |
| Very and fairly good | -0.063^**^ | [-0.109,-0.017] | -0.088^*^ | [-0.160,-0.017] | -0.154^***^ | [-0.207,-0.102] | -0.183^***^ | [-0.237,-0.128] |
| Neither good nor poor | 0.078^*^ | [0.019,0.137] | 0.103^*^ | [0.017,0.189] | 0.052 | [-0.029,0.132] | 0.052 | [-0.028,0.132] |
| Fairly poor | 0.160^***^ | [0.087,0.232] | 0.209^***^ | [0.087,0.331] | 0.067 | [-0.032,0.166] | 0.064 | [-0.033,0.161] |
| **Long-term Health Conditions (%, no as reference)** |  |  |  |  |  |  |  |  |
| Yes | 0.018 | [-0.017,0.052] | 0.004 | [-0.041,0.050] | 0.077^**^ | [0.022,0.132] | 0.054 | [-0.001,0.109] |
| **Gender (%, female as reference)** |  |  |  |  |  |  |  |  |
| Male | 0.003 | [-0.034,0.039] | 0.025 | [-0.027,0.077] | 0.051 | [-0.007,0.109] | 0.100^**^ | [0.036,0.163] |
| **Age (%, under 64 as reference)** |  |  |  |  |  |  |  |  |
| 65 to 74 years old | 0.022 | [-0.078,0.122] | 0.043 | [-0.099,0.185] | -0.151 | [-0.327,0.025] | -0.025 | [-0.187,0.136] |
| 75 to 84 years old | 0.093 | [-0.033,0.220] | 0.184^*^ | [0.004,0.365] | 0.063 | [-0.159,0.285] | 0.308^**^ | [0.090,0.527] |
| 85 years old and older | 0.201^*^ | [0.029,0.372] | 0.216 | [-0.011,0.442] | 0.248 | [-0.037,0.533] | 0.215 | [-0.083,0.513] |
| **Race (%, others as reference)** |  |  |  |  |  |  |  |  |
| White | 0.134^***^ | [0.076,0.192] | 0.152^***^ | [0.078,0.227] | 0.275^***^ | [0.186,0.365] | 0.287^***^ | [0.196,0.378] |
| Mixed | 0.356^***^ | [0.227,0.485] | 0.415^***^ | [0.250,0.581] | 0.501^***^ | [0.317,0.686] | 0.350^***^ | [0.144,0.555] |
| Asian | 0.107^***^ | [0.047,0.166] | 0.129^***^ | [0.053,0.205] | 0.243^***^ | [0.154,0.333] | 0.256^***^ | [0.162,0.350] |
| Black | 0.179^***^ | [0.102,0.256] | 0.179^***^ | [0.078,0.280] | 0.342^***^ | [0.224,0.461] | 0.366^***^ | [0.251,0.481] |
| **Working status (%, other as reference)** |  |  |  |  |  |  |  |  |
| Full or part-time work | 0.052^*^ | [0.001,0.104] | 0.044 | [-0.024,0.112] | 0.148^***^ | [0.070,0.225] | 0.073 | [-0.010,0.156] |
| Full-time education | -0.016 | [-0.076,0.044] | -0.085^*^ | [-0.166,-0.003] | 0.023 | [-0.079,0.125] | -0.043 | [-0.150,0.065] |
| Unemployed | 0.038 | [-0.046,0.121] | 0.066 | [-0.033,0.165] | 0.115 | [-0.023,0.253] | 0.011 | [-0.133,0.155] |
| Retired | 0.006 | [-0.087,0.100] | -0.030 | [-0.153,0.093] | 0.132 | [-0.023,0.287] | -0.109 | [-0.262,0.044] |
| **Healthcare related variables** |  |  |  |  |  |  |  |  |
| Average NHS payment per registered patient | 0.019^***^ | [0.014,0.024] | 0.015^***^ | [0.008,0.022] | 0.020^***^ | [0.011,0.029] | 0.022^***^ | [0.015,0.030] |
| The percentage of quality outcome framework (QOF) points achieved | 0.059^**^ | [0.018,0.099] | 0.082^**^ | [0.029,0.136] | 0.004 | [-0.063,0.070] | 0.081^***^ | [0.036,0.126] |
| Total number of GP in full time equivalent, per 10,000 registered patients | -0.043 | [-0.136,0.051] | -0.077 | [-0.192,0.037] | 0.063 | [-0.076,0.203] | 0.043 | [-0.086,0.172] |
| The percentage of GPs whose primary medical qualification is from the UK (from non-UK areas as reference) | 0.004 | [-0.003,0.012] | 0.020^***^ | [0.010,0.029] | 0.021^**^ | [0.008,0.034] | 0.019^**^ | [0.006,0.032] |
| **IMD score quintile 2015 (1^st^ quintile (most deprived) as reference)** |  |  |  |  |  |  |  |  |
| 2^nd^ quintile | -0.143 | [-0.833,0.547] | -0.310 | [-1.221,0.601] | -0.537 | [-1.786,0.712] | -0.123 | [-1.353,1.106] |
| 3^rd^ quintile | 0.301 | [-0.406,1.007] | -0.234 | [-1.149,0.680] | -0.096 | [-1.359,1.166] | 0.141 | [-1.141,1.423] |
| 4^th^ quintile | -0.446 | [-1.194,0.301] | -0.737 | [-1.738,0.264] | -1.219 | [-2.599,0.161] | -1.077 | [-2.448,0.293] |
| 5^th^ quintile (least deprived) | -0.906^*^ | [-1.802,-0.010] | -1.169^*^ | [-2.328,-0.010] | -1.896^*^ | [-3.451,-0.342] | -1.301 | [-2.912,0.309] |

ME, marginal effects; 95% confidence intervals in brackets; ^*^ *p* < 0.05, ^**^ *p* < 0.01, ^***^ *p* < 0.001.

**Supplementary Appendix Table S10 Subgroup analysis results: By low continuity**

| **Variables** | **Low continuity in the 1^st^ quartile**  **(N=8,692)** | | **Low continuity in the 2^nd^ quartile**  **(N=8,709)** | | **Low continuity in the 3^rd^ quartile**  **(N=8,819)** | | **Low continuity in the 4^th^ quartile**  **(N=8,905)** | |
| --- | --- | --- | --- | --- | --- | --- | --- | --- |
|  | **ME** | **95% CI** | **ME** | **95% CI** | **ME** | **95% CI** | **ME** | **95% CI** |
| **NEVER** | 2.896^***^ | [2.077,3.714] | 2.171^***^ | [1.113,3.229] | 2.175^***^ | [1.273,3.077] | 1.954^***^ | [1.537,2.371] |
| **Appointment experience (%, very poor as reference)** |  |  |  |  |  |  |  |  |
| Very and fairly good | -0.021 | [-0.053,0.011] | -0.082^***^ | [-0.119,-0.046] | -0.131^***^ | [-0.173,-0.088] | -0.266^***^ | [-0.314,-0.218] |
| Neither good nor poor | 0.093^***^ | [0.051,0.135] | 0.054^*^ | [0.005,0.103] | 0.039 | [-0.017,0.095] | -0.062 | [-0.137,0.013] |
| Fairly poor | 0.110^***^ | [0.062,0.159] | 0.096^**^ | [0.037,0.155] | 0.079^*^ | [0.006,0.152] | 0.095^*^ | [0.005,0.184] |
| **Long-term Health Conditions (%, no as reference)** |  |  |  |  |  |  |  |  |
| Yes | 0.023^**^ | [0.006,0.040] | 0.041^**^ | [0.016,0.066] | 0.051^**^ | [0.018,0.083] | 0.075^**^ | [0.023,0.127] |
| **Gender (%, female as reference)** |  |  |  |  |  |  |  |  |
| Male | -0.018 | [-0.038,0.002] | 0.010 | [-0.019,0.038] | 0.046^*^ | [0.010,0.081] | 0.012 | [-0.047,0.072] |
| **Age (%, under 64 as reference)** |  |  |  |  |  |  |  |  |
| 65 to 74 years old | 0.017 | [-0.035,0.070] | -0.050 | [-0.126,0.027] | -0.049 | [-0.151,0.053] | -0.014 | [-0.174,0.147] |
| 75 to 84 years old | -0.012 | [-0.079,0.054] | 0.081 | [-0.012,0.175] | 0.141^*^ | [0.011,0.271] | 0.190 | [-0.009,0.389] |
| 85 years old and older | 0.127^**^ | [0.039,0.214] | 0.124^*^ | [0.006,0.242] | 0.165 | [-0.006,0.336] | 0.472^***^ | [0.206,0.738] |
| **Race (%, others as reference)** |  |  |  |  |  |  |  |  |
| White | 0.045^**^ | [0.017,0.073] | 0.160^***^ | [0.118,0.201] | 0.238^***^ | [0.182,0.294] | 0.409^***^ | [0.320,0.499] |
| Mixed | 0.146^***^ | [0.079,0.213] | 0.330^***^ | [0.237,0.423] | 0.398^***^ | [0.274,0.522] | 0.693^***^ | [0.499,0.888] |
| Asian | 0.033^*^ | [0.004,0.061] | 0.144^***^ | [0.102,0.186] | 0.191^***^ | [0.132,0.249] | 0.366^***^ | [0.275,0.458] |
| Black | 0.080^***^ | [0.041,0.119] | 0.225^***^ | [0.170,0.280] | 0.305^***^ | [0.229,0.381] | 0.466^***^ | [0.352,0.579] |
| **Working status (%, other as reference)** |  |  |  |  |  |  |  |  |
| Full or part-time work | 0.040^**^ | [0.014,0.066] | 0.051^**^ | [0.013,0.089] | 0.080^**^ | [0.032,0.128] | 0.180^***^ | [0.104,0.257] |
| Full-time education | -0.018 | [-0.047,0.011] | -0.050^*^ | [-0.097,-0.002] | -0.004 | [-0.063,0.055] | 0.063 | [-0.046,0.173] |
| Unemployed | 0.045^*^ | [0.003,0.087] | 0.071^*^ | [0.010,0.133] | 0.059 | [-0.022,0.140] | 0.201^**^ | [0.068,0.335] |
| Retired | 0.025 | [-0.021,0.072] | -0.012 | [-0.078,0.055] | 0.002 | [-0.090,0.093] | 0.030 | [-0.120,0.180] |
| **Healthcare related variables** |  |  |  |  |  |  |  |  |
| Average NHS payment per registered patient | 0.010^***^ | [0.008,0.012] | 0.020^***^ | [0.016,0.023] | 0.018^***^ | [0.011,0.026] | 0.041^***^ | [0.032,0.050] |
| The percentage of quality outcome framework (QOF) points achieved | 0.035^***^ | [0.016,0.054] | 0.019 | [-0.008,0.045] | 0.020 | [-0.015,0.056] | 0.053^*^ | [0.005,0.102] |
| Total number of GP in full time equivalent, per 10,000 registered patients | -0.014 | [-0.066,0.038] | 0.005 | [-0.059,0.069] | -0.085 | [-0.176,0.006] | -0.072 | [-0.206,0.062] |
| The percentage of GPs whose primary medical qualification is from the UK (from non-UK areas as reference) | 0.005^**^ | [0.002,0.009] | 0.014^***^ | [0.008,0.019] | 0.016^***^ | [0.008,0.023] | 0.025^***^ | [0.013,0.036] |
| **IMD score quintile 2015 (1^st^ quintile (most deprived) as reference)** |  |  |  |  |  |  |  |  |
| 2^nd^ quintile | -0.041 | [-0.360,0.277] | 0.078 | [-0.409,0.565] | 0.336 | [-0.427,1.099] | -0.400 | [-1.580,0.780] |
| 3^rd^ quintile | 0.349 | [-0.005,0.703] | -0.167 | [-0.670,0.336] | 0.240 | [-0.505,0.986] | 0.243 | [-0.921,1.406] |
| 4^th^ quintile | 0.264 | [-0.134,0.662] | -0.239 | [-0.796,0.317] | -0.705 | [-1.512,0.103] | -1.810^**^ | [-3.016,-0.603] |
| 5^th^ quintile (least deprived) | 0.053 | [-0.399,0.504] | -0.669^*^ | [-1.323,-0.016] | -1.389^**^ | [-2.323,-0.454] | -2.382^***^ | [-3.768,-0.995] |

ME, marginal effects; 95% confidence intervals in brackets; ^*^ *p* < 0.05, ^**^ *p* < 0.01, ^***^ *p* < 0.001.

**Supplementary Appendix Table S11.1 Subgroup analysis results: By IMD score (1^st^ to 3^rd^ quintiles, to be continued)**

| **Variables** | **IMD 1^st^ quintile**  **(N=5,513)** | | **IMD 2^nd^ quintile**  **(N=6,938)** | | **IMD 3^rd^ quintile**  **(N=7,228)** | |
| --- | --- | --- | --- | --- | --- | --- |
|  | **ME** | **95% CI** | **ME** | **95% CI** | **ME** | **95% CI** |
| **NEVER** | 1.363^***^ | [1.017,1.710] | 1.378^***^ | [1.108,1.649] | 1.509^***^ | [1.251,1.766] |
| **Appointment experience (%, very poor as reference)** |  |  |  |  |  |  |
| Very and fairly good | -0.234^***^ | [-0.299,-0.169] | -0.169^***^ | [-0.220,-0.118] | -0.132^***^ | [-0.179,-0.086] |
| Neither good nor poor | -0.031 | [-0.113,0.051] | 0.047 | [-0.017,0.111] | 0.033 | [-0.028,0.095] |
| Fairly poor | 0.021 | [-0.080,0.122] | 0.040 | [-0.047,0.126] | 0.139^***^ | [0.063,0.214] |
| **Long-term Health Conditions (%, no as reference)** |  |  |  |  |  |  |
| Yes | 0.045^*^ | [0.001,0.089] | 0.037^*^ | [0.000,0.074] | 0.009 | [-0.027,0.045] |
| **Gender (%, female as reference)** |  |  |  |  |  |  |
| Male | 0.004 | [-0.049,0.058] | 0.011 | [-0.031,0.054] | -0.005 | [-0.046,0.036] |
| **Age (%, under 64 as reference)** |  |  |  |  |  |  |
| 65 to 74 years old | 0.008 | [-0.115,0.131] | 0.001 | [-0.097,0.100] | 0.024 | [-0.081,0.129] |
| 75 to 84 years old | -0.036 | [-0.182,0.111] | 0.028 | [-0.098,0.154] | 0.215^***^ | [0.089,0.341] |
| 85 years old and older | 0.175 | [-0.011,0.361] | 0.151 | [-0.007,0.310] | 0.329^***^ | [0.157,0.500] |
| **Race (%, others as reference)** |  |  |  |  |  |  |
| White | 0.277^***^ | [0.175,0.378] | 0.327^***^ | [0.253,0.402] | 0.279^***^ | [0.213,0.345] |
| Mixed | 0.352^***^ | [0.150,0.554] | 0.501^***^ | [0.349,0.654] | 0.493^***^ | [0.349,0.638] |
| Asian | 0.309^***^ | [0.182,0.437] | 0.309^***^ | [0.226,0.391] | 0.269^***^ | [0.197,0.342] |
| Black | 0.321^**^ | [0.106,0.535] | 0.556^***^ | [0.415,0.697] | 0.379^***^ | [0.270,0.487] |
| **Working status (%, other as reference)** |  |  |  |  |  |  |
| Full or part-time work | 0.143^***^ | [0.067,0.218] | 0.019 | [-0.042,0.079] | 0.057^*^ | [0.003,0.111] |
| Full-time education | 0.039 | [-0.051,0.129] | -0.126^***^ | [-0.198,-0.054] | -0.081^**^ | [-0.141,-0.021] |
| Unemployed | 0.150 | [-0.003,0.303] | 0.116^*^ | [0.006,0.226] | 0.087 | [-0.011,0.185] |
| Retired | 0.065 | [-0.044,0.174] | -0.005 | [-0.096,0.086] | -0.052 | [-0.148,0.044] |
| **Healthcare related variables** |  |  |  |  |  |  |
| Average NHS payment per registered patient | 0.020^***^ | [0.014,0.026] | 0.021^***^ | [0.016,0.025] | 0.021^***^ | [0.016,0.026] |
| The percentage of quality outcome framework (QOF) points achieved | 0.038 | [-0.016,0.091] | 0.013 | [-0.022,0.047] | 0.024 | [-0.010,0.058] |
| Total number of GP in full time equivalent, per 10,000 registered patients | 0.063 | [-0.057,0.184] | -0.148^***^ | [-0.237,-0.060] | -0.005 | [-0.101,0.090] |
| The percentage of GPs whose primary medical qualification is from the UK (from non-UK areas as reference) | 0.009 | [-0.002,0.021] | 0.009^*^ | [0.001,0.018] | 0.012^**^ | [0.004,0.020] |

ME, marginal effects; 95% confidence intervals in brackets; ^*^ *p* < 0.05, ^**^ *p* < 0.01, ^***^ *p* < 0.001.

**Supplementary Appendix Table S11.2 Subgroup analysis results: By IMD score (4^th^ and 5^th^ quintiles)**

| **Variables** | **IMD 4^th^ quintile**  **(N=7,815)** | | **IMD 5^th^ quintile**  **(N=7,631)** | |
| --- | --- | --- | --- | --- |
|  | **ME** | **95% CI** | **ME** | **95% CI** |
| **NEVER** | 1.153^***^ | [0.916,1.389] | 1.109^***^ | [0.904,1.313] |
| **Appointment experience (%, very poor as reference)** |  |  |  |  |
| Very and fairly good | -0.137^***^ | [-0.173,-0.101] | -0.055^***^ | [-0.082,-0.028] |
| Neither good nor poor | 0.014 | [-0.035,0.064] | 0.067^**^ | [0.027,0.108] |
| Fairly poor | 0.043 | [-0.021,0.107] | 0.099^***^ | [0.049,0.149] |
| **Long-term Health Conditions (%, no as reference)** |  |  |  |  |
| Yes | 0.064^***^ | [0.034,0.095] | 0.053^***^ | [0.029,0.077] |
| **Gender (%, female as reference)** |  |  |  |  |
| Male | 0.015 | [-0.019,0.050] | 0.001 | [-0.026,0.028] |
| **Age (%, under 64 as reference)** |  |  |  |  |
| 65 to 74 years old | -0.082 | [-0.189,0.025] | -0.013 | [-0.104,0.078] |
| 75 to 84 years old | 0.130 | [-0.005,0.266] | 0.098 | [-0.024,0.219] |
| 85 years old and older | 0.184^*^ | [0.007,0.361] | 0.202^*^ | [0.037,0.367] |
| **Race (%, others as reference)** |  |  |  |  |
| White | 0.162^***^ | [0.111,0.213] | 0.109^***^ | [0.077,0.141] |
| Mixed | 0.315^***^ | [0.203,0.427] | 0.251^***^ | [0.171,0.331] |
| Asian | 0.128^***^ | [0.076,0.181] | 0.085^***^ | [0.054,0.115] |
| Black | 0.186^***^ | [0.123,0.249] | 0.155^***^ | [0.114,0.196] |
| **Working status (%, other as reference)** |  |  |  |  |
| Full or part-time work | 0.098^***^ | [0.051,0.145] | 0.052^**^ | [0.020,0.083] |
| Full-time education | 0.043 | [-0.011,0.096] | -0.004 | [-0.055,0.046] |
| Unemployed | 0.167^***^ | [0.091,0.243] | 0.014 | [-0.035,0.064] |
| Retired | 0.060 | [-0.037,0.157] | -0.062 | [-0.151,0.027] |
| **Healthcare related variables** |  |  |  |  |
| Average NHS payment per registered patient | 0.021^***^ | [0.014,0.028] | 0.018^***^ | [0.011,0.024] |
| The percentage of quality outcome framework (QOF) points achieved | 0.052^**^ | [0.016,0.088] | 0.042^**^ | [0.016,0.067] |
| Total number of GP in full time equivalent, per 10,000 registered patients | -0.050 | [-0.141,0.041] | 0.015 | [-0.056,0.086] |
| The percentage of GPs whose primary medical qualification is from the UK (from non-UK areas as reference) | 0.020^***^ | [0.013,0.027] | 0.012^***^ | [0.007,0.017] |

ME, marginal effects; 95% confidence intervals in brackets; ^*^ *p* < 0.05, ^**^ *p* < 0.01, ^***^ *p* < 0.001.
