# Supplementary material for: Less continuity with more complaints: a repeated cross-sectional study of the association between relational continuity of care and patient complaints in English general practice
Source: BMJ Qual Saf. 2025 Oct 7;35(6):e018989. doi: 10.1136/bmjqs-2025-018989 (PMC13217021; doi:10.1136/bmjqs-2025-018989)
Supplement: online supplemental file 7 [file bmjqs-35-6-s007.docx]

**Supplementary Appendix Table S12 Mediation analysis results: outcome model**

| **Variables** | **Marginal Effects** | **SE** | **95% CI** | **P values** | **Marginal Effects** | **SE** | **95% CI** | **P values** |
| --- | --- | --- | --- | --- | --- | --- | --- | --- |
| NEVER | 1.645 | (0.087) | [1.474,1.815] | 0.000 | 1.685 | (0.105) | [1.480,1.891] | 0.000 |
| Lost trust | 0.195 | (0.022) | [0.153,0.238] | 0.000 |  |  |  |  |
| NEVER * Lost trust | -0.073 | (0.010) | [-0.093,-0.053] | 0.000 |  |  |  |  |
| Unmet needs |  |  |  |  | -0.061 | (0.011) | [-0.082,-0.041] | 0.000 |
| NEVER * Unmet needs |  |  |  |  | 0.188 | (0.023) | [0.143,0.233] | 0.000 |
| N | 34908 |  |  |  | 29323 |  |  |  |

Note: The two outcome models were fitted with the Negative Binomial model with adjustment for all confounders as same as in the baseline regression. In both models, the total number of new complaints per 10,000 patients was the outcome of interest.

**Supplementary Appendix Table S13 Mediation analysis results: mediator model**

| **Outcome** | **Exposure** | **Coefficients** | **SE** | **95% CI** | **P values** | **N** |
| --- | --- | --- | --- | --- | --- | --- |
| Lost trust | NEVER | 0.377 | (0.025) | [0.329,0.425] | 0.000 | 34908 |
| Unmet needs |  | 0.373 | (0.028) | [0.319,0.428] | 0.000 | 29323 |

Note: The mediator models were fitted with linear regression model, with ‘the percentage of patients who NEVER see their preferred GP (NEVER)’ as the main independent variable and ‘lost trust’ and ‘unmet needs’ as the outcomes of interests separately.

**Supplementary Appendix Table S14 Mediation analysis results: with and without adjusting for the mediators**

| **Outcome** | **Exposure** | **Negative Binomial Models** | **Marginal Effects** | **SE** | **95% CI** | **P values** | **N** |
| --- | --- | --- | --- | --- | --- | --- | --- |
| Complaints | NEVER | Multivariable | 1.343 | (0.059) | [1.227,1.458] | 0.000 | 35125 |
|  |  | Multivariable + Lost trust | 1.172 | (0.059) | [1.057,1.288] | 0.000 | 34908 |
|  |  | Multivariable + Unmet needs | 1.194 | (0.066) | [1.064,1.324] | 0.000 | 29323 |
|  |  | Multivariable + Lost trust + Unmet needs | 1.197 | (0.067) | [1.067,1.328] | 0.000 | 29158 |
