# Supplementary material for: Less continuity with more complaints: a repeated cross-sectional study of the association between relational continuity of care and patient complaints in English general practice
Source: BMJ Qual Saf. 2025 Oct 7;35(6):e018989. doi: 10.1136/bmjqs-2025-018989 (PMC13217021; doi:10.1136/bmjqs-2025-018989)
Supplement: online supplemental file 8 [file bmjqs-35-6-s008.docx]

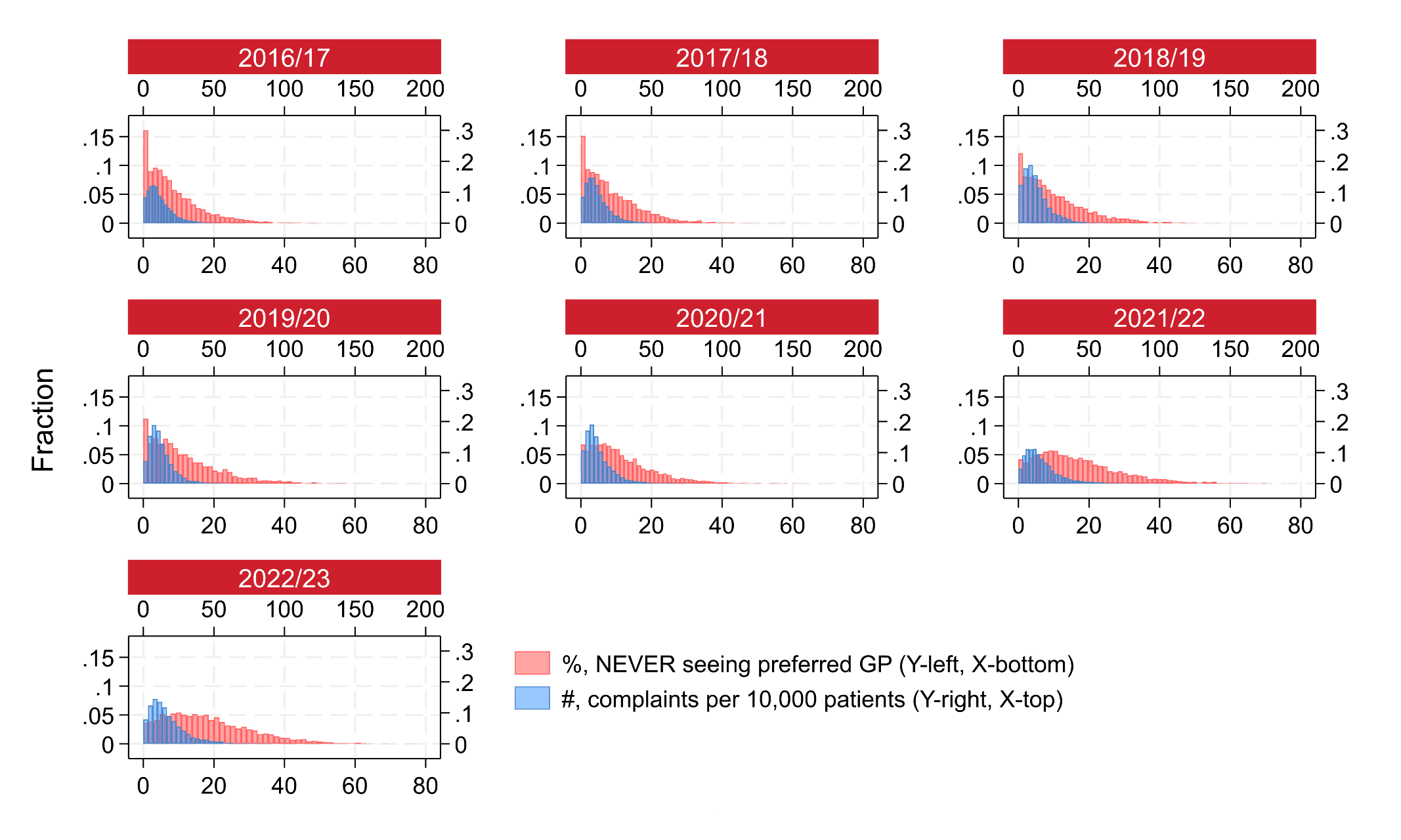


Supplementary Figure S1. The distribution of discontinuity of care and patient complaints

Notes:

(1) ‘%, NEVER seeing preferred GP’ is the main independent variable, which is measured by the proportion of patients who NEVER seeing their preferred GP.

(2) ‘#, complaints per 10,000 patients’ is the outcome, the number of total new written complaints per 10,000 patients at practice level.

(3) For the ‘**NEVER**’ variable, its **Y-axis** is on the **left** (the fraction of practices with specific proportion of patients NEVER seeing their preferred GP) and **X-axis** is on the **bottom** (the practice level proportion of patients NEVER seeing their preferred GP).

(4) For the ‘**complaints**’ variable, its **Y-axis** is on the **right** (the fraction of practices with specific number of total new complaints per 10,000 patients) and **X-axis** is on the **top** (the practice level number of total new complaints per 10,000 patients)
